# Quantitative evaluation of skin pigmentation effects on photoplethysmography using vascular finger phantoms and Monte Carlo simulation

**DOI:** 10.1117/1.JBO.31.8.087001

**Published:** 2026-08-22

**Authors:** Laura Osorio-Sanchez, James M. May, Panicos A. Kyriacou

**Affiliations:** City St. George’s, University of London, Research Centre for Biomedical Engineering, London, United Kingdom

**Keywords:** photoplethysmography, tissue phantoms, skin pigmentation, Monte Carlo modelling, light-tissue interaction

## Abstract

**Significance:**

Photoplethysmography (PPG) underpins a wide range of clinical and consumer optical monitoring technologies. However, differences in skin pigmentation can alter light–tissue interactions through increased absorption by melanin, affecting PPG signal quality and potentially contributing to performance disparities across populations.

**Aim:**

This study aimed to investigate the effect of skin pigmentation on PPG signal quality by combining an advanced vascular finger phantom with Monte Carlo simulations to directly link experimentally measured PPG features to underlying photon transport behavior in both reflectance and transmittance measurement modes.

**Approach:**

A multilayer vascular finger phantom incorporating a three-level, physiologically inspired vascular network and interchangeable skin layers representing pale, medium, and dark skin pigmentation was developed. The optical properties were characterized across 530, 665, and 940 nm. PPG signals were acquired in reflectance and transmittance modes under controlled pulsatile flow conditions. Quantitative signal metrics, including peak-to-peak amplitude, signal-to-noise ratio (SNR), and AC/DC ratio, were extracted. Monte Carlo simulations were implemented using the measured optical properties to model photon transport, energy deposition, and depth-dependent absorption.

**Results:**

Increasing skin pigmentation resulted in systematic reduction in all PPG features across all wavelengths, with the strongest attenuation observed at shorter wavelengths. Monte Carlo simulations revealed increased superficial absorption and reduced photon penetration in darker skin layers, closely matching experimental trends.

**Conclusions:**

This combined experimental-computational framework provides quantitative and mechanistic insight into how skin pigmentation influences light–tissue interaction and PPG signal quality, with direct relevance to emerging wearables and PPG-based applications, where signal quality disparities across skin tones may impact algorithm performance.

## 1 Introduction

Photoplethysmography (PPG) forms the basis of many optical monitoring technologies used in both clinical practice and consumer health devices. Although the influence of skin pigmentation on the accuracy of pulse oximetry and related light–tissue interaction methods was first discussed more than four decades ago,^1^^–^^5^ recent clinical evidence has brought renewed attention to this issue. In a large retrospective study, Sjoding et al.^6^ reported systematic differences in pulse oximetry performance between Black and White patients, with Black patients experiencing nearly three times the frequency of undetected hypoxemia under hypoxic conditions. Such findings have reinforced concerns that skin pigmentation may alter optical signals. Given the widespread use of PPG-based techniques, there is a need to improve the physical understanding of how skin pigmentation-related absorption and scattering influence PPG signals and potentially contribute to observed disparities.

Understanding how skin pigmentation influences PPG signal formation is challenging in *in vivo* studies, where optical effects are coupled with physiological variability such as perfusion, tissue composition, and vascular dynamics.^7^ As a result, it is difficult to isolate the contribution of pigmentation-related optical properties from other confounding factors. In vitro tissue phantoms offer a controlled alternative, enabling systematic investigation of light–tissue interactions under reproducible conditions. When combined with Monte Carlo simulations of photon transport, phantom-based studies provide a powerful approach for linking PPG signals to underlying optical energy deposition, photon path distributions, and absorption profiles.

Controlled experimental approaches have previously been used to investigate PPG signal formation using optical tissue phantoms, primarily in reflectance configurations.^8^^–^^13^ Rodriguez et al. used an anatomically inspired optical finger phantom to evaluate PPG signal formation under controlled conditions with an embedded vascular network.^8^ Similarly, Chen et al. fabricated a multilayer phantom with tunable optical properties across the visible and near-infrared spectrum, providing a well-characterized tool for optical validation.^9^ Bhusal et al. evaluated a pulsatile, channel-based finger phantom and demonstrated that epidermal melanin layers influenced reflectance PPG waveforms.^10^ Jenne and Zappe developed a multilayer tissue phantom with expandable vascular structures that replicate the optical and mechanical properties relevant to PPG signal.^11^ Previous work from the Research Centre for Biomedical Engineering (RCBE) similarly designed and fabricated a multilayer vascular finger phantom to investigate the effect of skin pigmentation on reflectance PPG signals.^12^ Despite these advances, existing phantom-based studies have predominantly focused on reflectance-only measurement geometries, have often relied on simplified representations of vascular anatomy, and have not linked observations to depth photon transport metrics.

In parallel to these experimental efforts, Monte Carlo models have been widely used to investigate light transport in tissue and to assess how variations in optical properties, including melanin concentrations, influence photon propagation and detected signals. These simulations have provided valuable insights into depth sensitivity and absorption behavior across tissue layers.^14^^–^^17^ However, Monte Carlo approaches have largely been applied independently of phantom-based experiments and often have not been integrated with *in vitro* studies for the evaluation of skin pigmentation effects on PPG signals. As a result, the combined influence of skin pigmentation, physiologically representative vascular network, and photon transport on PPG signal formation remains incompletely characterized.

To address these gaps, this study combines an advanced multilayer vascular finger phantom with Monte Carlo simulations to investigate the effect of skin pigmentation on PPG signals in both reflectance and transmittance measurement modes. By directly linking experimentally measured PPG signals to depth-dependent photon transport metrics, this work aims to improve understanding of how skin pigmentation-related optical properties influence light–tissue interaction and PPG quality.

## 2 Methodology

Building upon our previous work on a vascular finger phantom with interchangeable skin-tone layers,^12^ an advanced version of the phantom was designed and developed to enhance the overall perfusion and physiological realism of the model. The initial phantom demonstrated that limited perfusion within the vascular channel restricted the PPG signal’s formation, resulting in poor signal quality or the absence of green-light PPG in medium and dark skin tones, and no transmittance mode signals were observed. In addition to the restricted perfusion, other factors such as the relatively thick skin layer, the deep positioning of the vessel (∼2.5  mm from the surface), and the high scattering of the adipose layer further attenuated the optical signal and limited light penetration, particularly at shorter wavelengths. To overcome these challenges, the vascular network of the phantom was redesigned with a channel-based perfusion network that aimed to enhance fluid distribution throughout the fingertip and promote more uniform pulsatile flow. This new configuration improves light–tissue interaction, enabling the acquisition of both reflectance and transmittance PPG signals across all skin tones and relevant wavelengths.

### 2.1 Finger Phantom

#### 2.1.1 Finger phantom design

The advanced finger phantom preserved the multilayer architecture of the previous version, consisting of a subcutaneous adipose matrix, an interchangeable skin layer, and a vascularization-simulating channel network. Together, these components replicate the anatomical, optical, and mechanical features of the human index finger, with particular emphasis on enhancing fluid perfusion.

The vascular network design was guided by the arterial anatomy of the fingertip. In the human finger, two proper palmar digital arteries converge distally to form the superficial arcade, giving rise to longitudinal branches that create the proximal and distal subungual arcades.^18^^–^^20^ To approximate this behavior within fabrication constraints, a simplified single-inlet, single-outlet network was implemented that bifurcated into multiple channels, simulating the superficial and subungual arcades. Although the model did not replicate venous drainage, the branching pattern provided physiologically relevant fluid dynamics suitable for dynamic PPG signal generation.

**Fig. 1:**
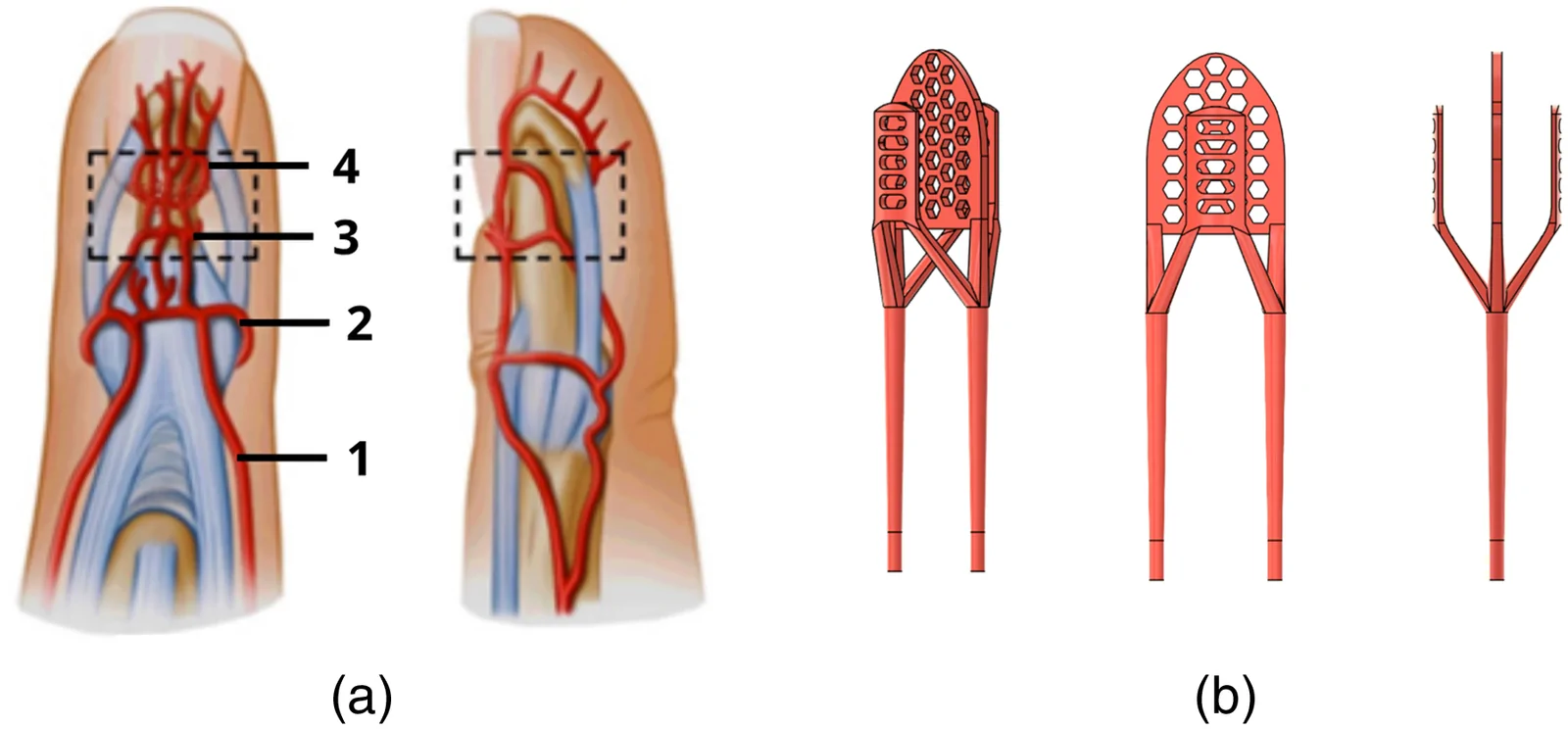
(a) Schematic of the arterial anatomy of the human fingertip, highlighting the superficial and subungual arcades (adapted from Jasionyte et al.^20^). (b) Simplified three-layer vascular channel geometry implemented in the finger phantom, representing superficial and deep perfusion pathways within fabrication constrains.

The vascular geometry consisted of three interconnected channel layers (Fig. 1). The two outer layers, representing superficial perfusion near the dermal surface, comprised parallel oval-shaped channels with a radius of 0.7 mm, a spacing of 0.6 mm, and a layer thickness of 1 mm, arranged to follow the curved fingertip contour. The inner layer simulated deeper perfusion within the fingertip core and consisted of a 1 mm thick honeycomb structure formed by 0.9 mm hexagonal cells separated by 0.7 mm spacing. Oblique perforator channels connected the outer and inner layers, enabling volumetric fluid exchange between peripheral and central regions and allowing distributed pulsatile flow across multiple depths.

**Fig. 2:**
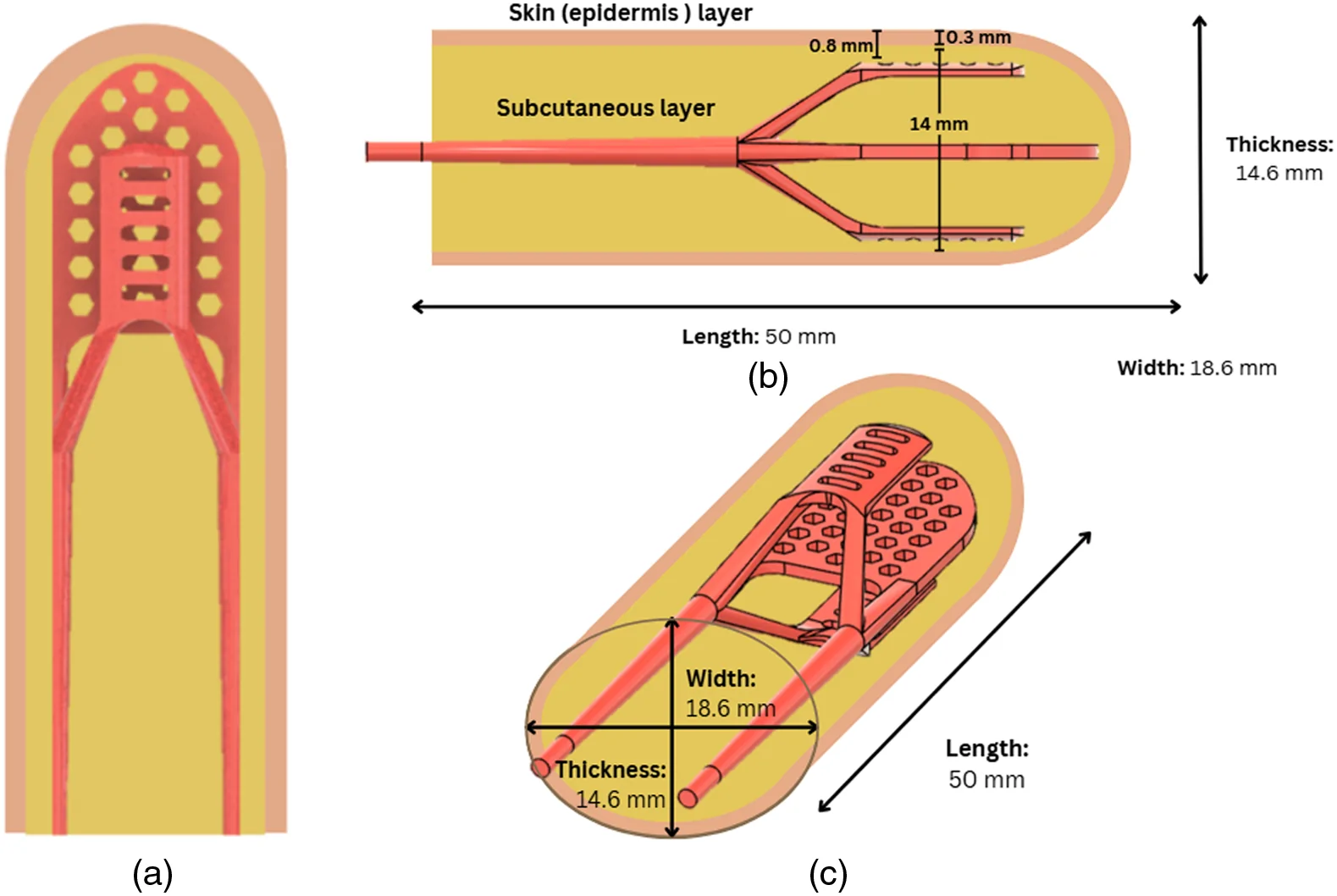
Schematic design and dimensions of the advanced finger (a) Top view showing the multilayer structure and embedded vascular network within the adipose matrix. (b) Longitudinal cross-section illustrating skin thickness, subcutaneous tissue, and depth of the vascular channels. (c) Perspective view of the phantom highlighting overall geometry.

Figure 2 illustrates the schematic design and dimensions of the advanced finger phantom. Only the distal and middle phalanges of the index finger were replicated, corresponding to a total length of 50 mm, a width of 18.6 mm, and a thickness of 14.6 mm, consistent with anatomical averages reported in the literature.^21^ The model consisted of three layers: (1) an outer skin layer representing the epidermis, with a thickness of 0.3 mm; (2) a subcutaneous adipose layer forming the bulk matrix; and (3) an embedded three-layer vascular channel network. The skin layer was designed to match reported ranges for human volar epidermal fingertip thickness (0.1 to 0.5 mm),^22^^–^^26^ ensuring optical and mechanical realism. The vascular network occupied approximately the distal 14 mm of the phantom and was positioned with its most superficial channels ∼0.8  mm below the skin surface, enabling sensitivity to both reflectance and transmittance PPG illumination geometries.

#### 2.1.2 Optical and mechanical characterization

To ensure optical realism, the optical properties of the phantom layers were tailored to replicate the absorption and scattering characteristics of human skin and subcutaneous tissue across 500 to 1000 nm wavelength range. All formulations were based on Platsil Gel-00 silicone (Polytek Development Corp., Easton, Pennsylvania, United States), selected for its refractive index (n=1.4) comparable to that of human soft tissue. Optical properties were tuned by adjusting the concentrations of titanium dioxide (${\mathrm{TiO}}_{2}$; Kronos Worldwide Inc., Dallas, Texas, United States) as the scattering agent and a combination of Bismarck Brown (BDH Chemicals Ltd., London, England), India Ink (Jackson’s Art Supplies, London, United Kingdom), and Epolight 5532 dye (Epolin Inc., Newark, New Jersey, United States) as absorbers.

Three skin tone layers representing pale, medium, and dark pigmentation were fabricated, corresponding approximately to Fitzpatrick I to II, III to IV, and V to VI categories. The optical properties were adjusted by varying absorber concentrations while maintaining constant scattering concentration. The formulations for both adipose and skin layers (Table 1) were iteratively refined to match the absorption ($\mu_{a}$) and reduced scattering ($\mu_{s}^{\prime }$) coefficients reported in the literature for human adipose tissue^27^^–^^31^ and skin across different pigmentation levels.^30^^,^^32^^,^^33^ Absorbers and the scatterer were first mixed with Part A silicone and the curing retarder, followed by the addition of Part B at an equal ratio. The mixture was then thoroughly stirred and degassed under vacuum to eliminate air bubbles.

**Table 1:** Concentrations of absorbing and scattering agents are used in the formulations of adipose and skin tone layers. Volumes are reported as microliters or milligrams of stock solution added per gram of silicone mix. Stock solution concentrations are given in parentheses.

Layer	Bismarck Brown stock solution (25 mg/L in isopropanol) [μL/g]	Epolight dye stock solution (1 mg/mL in acetone) [μL/g]	India ink (1% v/v) [μL/g]	Titanium dioxide [mg/g]
Adipose	7.74	0	0	0.5
Pale skin tone	20	70	10	1
Medium skin tone	40	100	20	1
Dark skin tone	130	400	30	1

Optical characterization was performed using a Lambda 1050 dual-beam spectrophotometer (Perkin Elmer Corp., Waltham, Massachusetts, United States) equipped with a 100 mm integrating sphere and PMT/InGaAs detector. Total reflectance and transmittance were measured over the 500 to 1000 nm range using 10 mm path-length cuvettes. The $\mu_{a}$ and $\mu_{s}^{\prime }$ were then extracted using the Inverse Adding-Doubling (IAD) algorithm,^34^ assuming a refractive index of 1.4 and anisotropy factor (g) of 0.9. These measured optical parameters were used as inputs for the subsequent Monte Carlo light-transport simulations (Table 2).

**Table 2:** Optical properties used in the Monte Carlo simulations for each tissue-simulating layer of the finger phantom at three wavelengths (530, 665, and 940 nm). The absorption coefficient ($\mu_{a}$) and reduced scattering coefficient ($\mu_{s}^{\prime }$) were obtained experimentally from the optical characterization of the fabricated finger phantom, while anisotropy factor (g), and refractive index (n) were assumed on typical values reported for soft tissue. The scattering coefficient ($\mu_{s}$) was calculated from $\mu_{s}^{\prime }$ and g.

Tissue layer / Component	$\mu_{a}$ $\left({\mathrm{mm}}^{-1}\right)$			$\mu_{s}^{\prime }$ $\left({\mathrm{mm}}^{-1}\right)$			$\mu_{\text{s}}$ $\left({\mathrm{mm}}^{-1}\right)$			g
Wavelength	530 nm	665 nm	940 nm	530 nm	665 nm	940 nm	530 nm	665 nm	940 nm	
Pale skin	0.183	0.053	0.022	1.612	2.249	1.427	16.120	22.490	14.270	0.900
Medium skin	0.248	0.092	0.034	1.296	2.050	1.402	12.964	20.497	14.020	0.900
Dark skin	0.290	0.134	0.067	1.018	1.603	1.291	10.180	16.030	12.910	0.900
Adipose	0.037	0.013	0.004	1.565	1.246	0.607	15.649	12.455	6.066	0.900
Blood	0.162	0.119	0.071	0.254	0.161	0.036	2.535	1.611	0.364	0.900

**Fig. 3:**
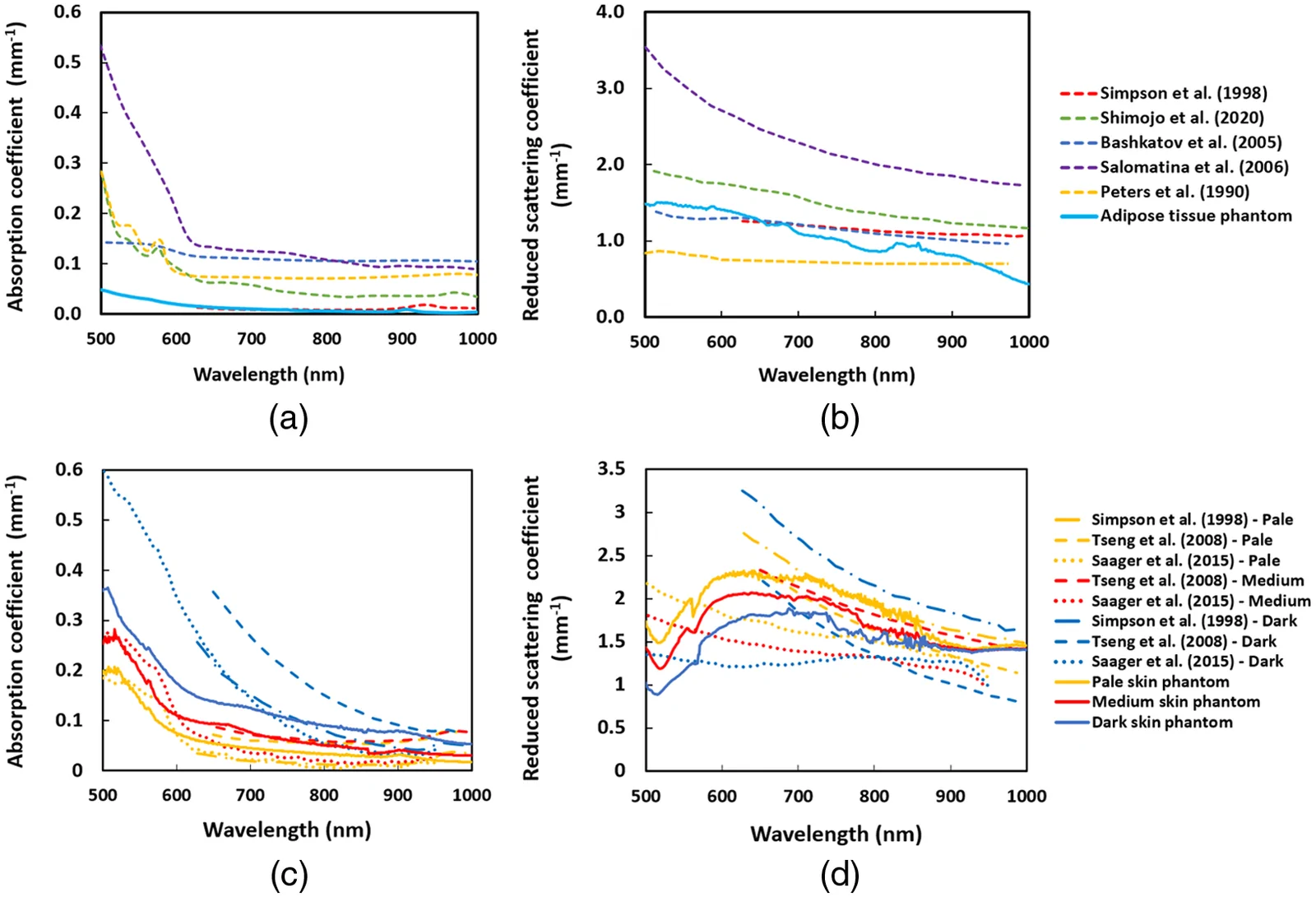
Measured absorption ($\mu_{a}$) and reduced scattering ($\mu_{s}^{\prime }$) coefficients of the adipose and skin-tone layers compared with literature data. Panels (a)–(b) show the adipose phantom versus reported values from Refs. 27–31. Panels (c)–(d) show pale, medium, and dark skin phantoms versus literature data from Refs. 30, 32, 33 within 500 to 1000 nm wavelength range.

The measured $\mu_{a}$ and $\mu_{s}^{\prime }$ spectra for the adipose and the three skin tones are shown in Fig. 3. The absorption coefficients have a strong dependence on pigmentation in the visible range, with higher $\mu_{a}$ values at 530 nm for darker tones due to higher melanin concentration. The reduced scattering shows a non-monotonic trend across wavelengths, decreasing from 500 to 530 nm, increasing toward 665 nm, and decreasing again at longer wavelengths, consistent with a general decrease toward the infrared.

In addition to optical characterization, the mechanical properties of the phantom materials were assessed to ensure physiologically relevant tissue compliance. Platsil Gel-00 silicone was selected due to its ease of handling, optical stability, and ability to reproduce human soft tissue. Target hardness values were guided by reported human fingertip measurements (25 to 35 Shore OO).^35^^,^^36^ Hardness was measured using a Shore OO durometer following ASTM D2240-15, with values reported as the mean of multiple measurements. The resulting hardness was 31.2±1.9 (Shore OO), falling within the physiological range.

#### 2.1.3 Finger phantom fabrication

**Fig. 4:**
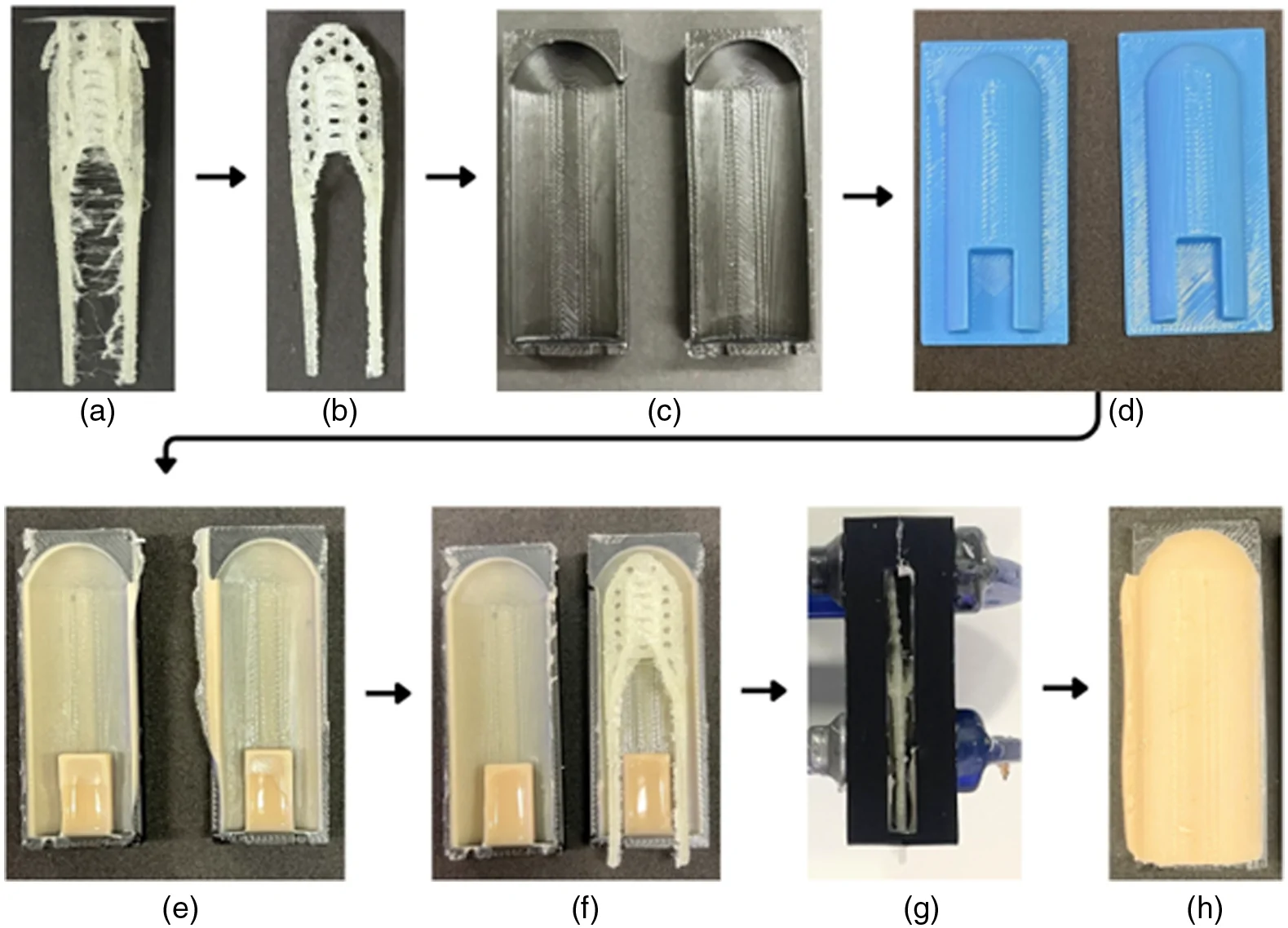
Fabrication of the finger phantom. (a) 3D-printed PVA sacrificial template. (b) Cleaned PVA structure, (c) Two-part finger mold. (d) Secondary mold for casting a 0.5 mm adipose silicone layer. (f) Placement of the PVA network within the mold. (g) Mold assembly and silicone casting. (h) Final phantom after curing and dissolution of the PVA.

The vascular channel network was fabricated and integrated into the finger phantom through a casting process (Fig. 4). The vascular network was 3D printed using a Bambu Lab X1 Carbon Series printer (Bambu Lab, Hong Kong, China) with polyvinyl alcohol (PVA) filament, allowing it to act as a water-soluble sacrificial template material [Fig. 4(a)]. After printing, the PVA structure was cleaned to remove supports and residual filament using fine tools. To prevent damage to the delicate structure, any remaining filaments were softened in warm water (approximately 40°C) and carefully detached [Fig. 4(b)].

The external finger geometry was formed using a custom two-part mold representing the distal and middle phalanges of an adult male index finger [Fig. 4(c)]. Before embedding the PVA network, a uniform 0.5 mm adipose silicone coating was fabricated using a secondary mold scaled down 0.5 mm on all sides [Fig. 4(d)], ensuring a consistent positioning of the vascular network ∼0.5  mm below the surface of the adipose region, corresponding to the depth of the superficial vascular plexus in vivo [Fig. 4(e)]. The PVA network was then placed within the mold cavity [Fig. 4(f)], after which the mold halves were assembled and sealed. The remaining adipose silicone mix was poured through the dedicated 4 mm inlet [Fig. 4(g)] and allowed to cure. One cured, the mold was removed [Fig. 4(h)], and the PVA template was dissolved in warm water, leaving a fully enclosed internal channel network.

#### 2.1.4 In vitro experimental setting and protocol

**Fig. 5:**
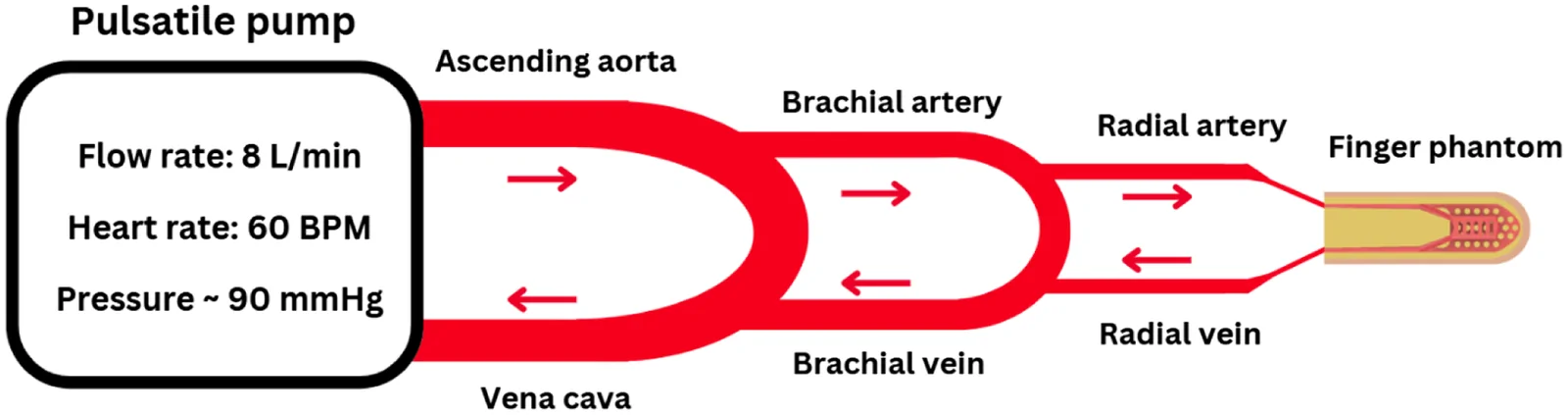
Schematic of the *in vitro* cardiovascular flow loop used to evaluate the vascular finger phantom. The programmable pulsatile pump generates physiological pulsatile flow that circulates through the ascending aorta, brachial artery, radial artery, and the finger phantom before returning through the venous pathway (radial vein, brachial vein, and vena cava) in a closed-loop circuit. The system was operated at a heart rate of 60 bpm, with a flow rate of 8 L/min, producing an overall system pressure of ∼90  mmHg.

The fabricated vascular finger phantom was evaluated using the *in vitro* cardiovascular system previously described.^12^ The system reproduces physiological pulsatile flow and pressure conditions throughout a closed-loop circuit driven by a programmable pulsatile pump (BDC Labs, Colorado, United States), connected to the phantom via compliant tubing that mimics the peripheral arterial tree (Fig. 5). During the experiments, the system was operated at a heart rate of 60 bpm with a flow rate of 8  L/min, generating an overall system pressure of ∼90  mmHg. A blood mimicking fluid composed of 0.1% w/v Nigrosine dye (Thermo Fisher Scientific, Loughborough, United Kingdom) diluted in deionized water was circulated through the vascular network to simulate the broadband optical absorption of blood across 500 to 1000 nm wavelength range.

Both reflectance and transmittance PPG configurations were implemented to assess the influence of the three pigmented skin layers on optical performance. A custom-made PPG finger clip probe operating in reflectance and transmittance mode was used. In the reflectance mode, the light source and the reflectance photodetector were positioned on the same side of the fingertip with a source-detector separation of 4 mm. In the transmittance mode, the LEDs were positioned directly opposite the transmittance photodetector, enabling light to pass through the fingertip. The PPG finger clip probe was interfaced with a PPG acquisition system developed by the Research Centre for Biomedical Engineering (RCBE),^37^ at a sampling rate of 2 kHz. The probe incorporated an integrated multiwavelength LED chip (SFH 7016, OSRAM), with all LEDs driven at a constant current of 40 mA. The manufacturer’s datasheet reports a total radiant flux values of 14, 14, and 11 mW at 20 mA, for the green, red, and infrared LEDs respectively, at 20 mA^38^ drive current, indicating comparable optical output across wavelengths at equivalent drive conditions. As all three LEDs were subject to the same drive current in the present study, the relative comparability of optical output across wavelengths is expected to be preserved.

PPG signals were recorded for five minutes per skin tone phantom under pulsatile conditions. Signal processing was performed in MATLAB (R2024a) using a fourth-order Butterworth band-pass filter (0.4 to 10 Hz) to extract the AC component and a low-pass filter (<0.3  Hz) for the DC component. The AC/DC ratio, signal-to-noise ratio (SNR), and amplitude were computed to quantify the impact of skin pigmentation on PPG signal quality.

In this study, the AC/DC ratio is used as a measure of relative pulsatile signal strength and signal quality, rather than within the conventional ratio-of-ratios formulation used in pulse oximetry. AC amplitude and AC/DC were calculated using peak-to-peak measurements of the pulsatile PPG waveform, while SNR was computed from 5-s analysis windows as the ratio of signal power within the physiological frequency band (0.5 to 10 Hz) to high-frequency power (>10  Hz), estimated using a high-pass filter. The calculation was implemented using MATLAB’s built-in snr function.

### 2.2 Monte Carlo Modelling of Photon-Tissue Interaction

Monte Carlo simulations were performed to model photon propagation through the multilayer structure of the vascular finger phantom and to quantify how skin pigmentation influences light transport and signal detection. This computational approach complements the *in vitro* experiments by isolating the contribution of tissue absorption and scattering to the measured PPG amplitudes.

#### 2.2.1 Model geometry and optical properties

**Fig. 6:**
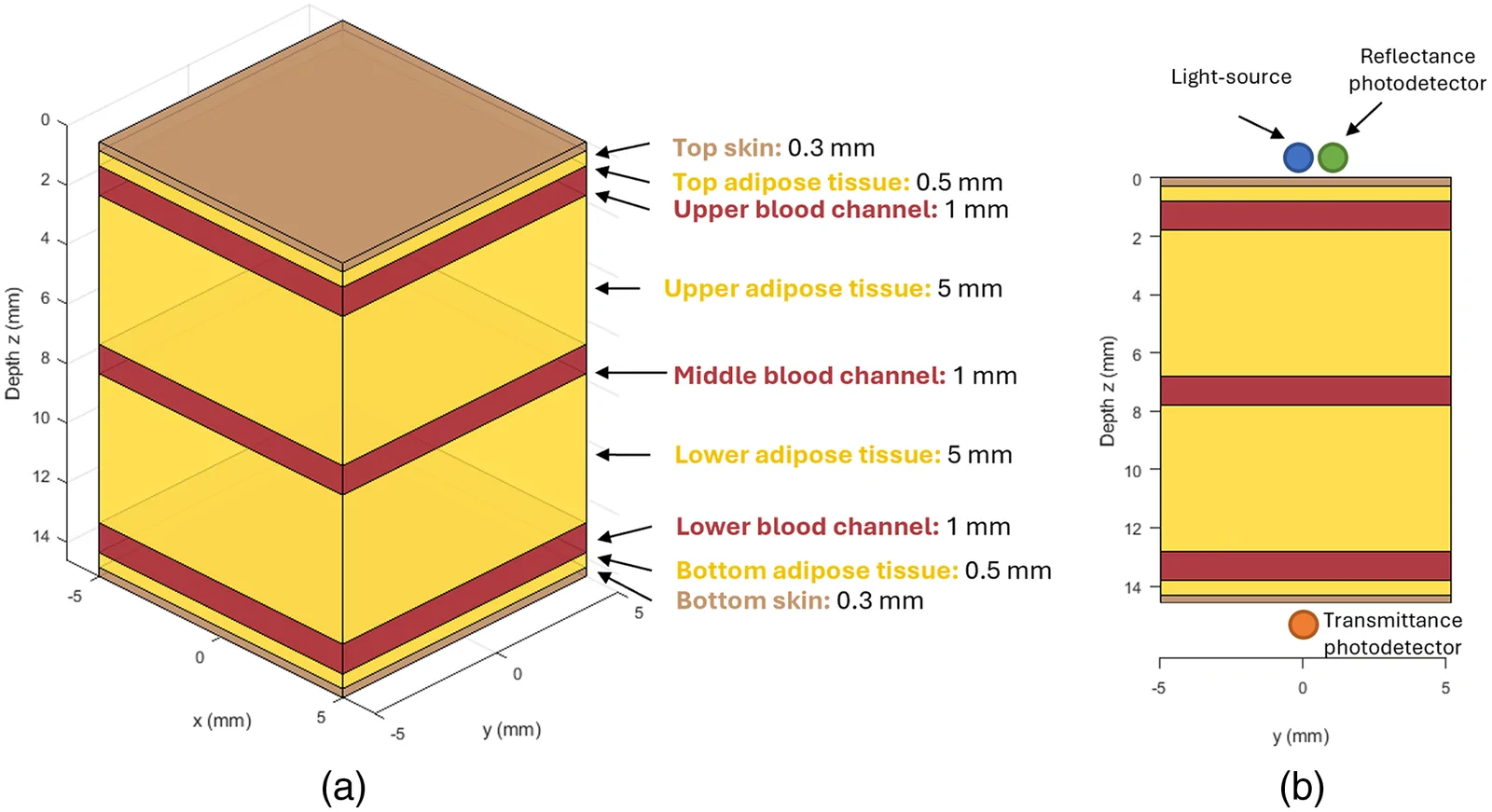
Geometry of the multilayer Monte Carlo model replicating the vascular finger phantom. (a) 3D schematic of the layered tissue geometry showing the top and bottom skin layers, adipose regions, and embedded blood channels. (b) Source–detector configuration used in reflectance and transmittance simulations, matching the *in vitro* PPG setup.

The Monte Carlo model was constructed based on the multi-layered tissue framework described by Wang and Jacques,^39^ representing the same geometry and layer arrangement used in the fabricated finger phantom. As shown in Fig. 6, the tissue volume consisted of three primary optical regions: skin, adipose tissue, and blood, arranged into multiple sublayers to replicate the spatial distribution of the vascular channels. The total tissue thickness was 14.6 mm. From top to bottom, the layers were defined as: top skin (0.3 mm), top adipose (0.5 mm), upper blood (1.0 mm), upper adipose (5.0 mm), middle blood (1.0 mm), lower adipose (5.0 mm), lower blood (1.0 mm), bottom adipose (0.5 mm), and bottom skin (0.3 mm). The three vascular layers were included to represent the depth distribution of the vascular network within the phantom. A schematic of the model geometry and source-detector configuration is shown in Fig. 6.

The optical properties assigned to each layer were derived directly from the experimental optical characterization ($\mu_{a}$ and $\mu_{s}^{\prime }$) of the phantom described in Sec. 2.1.2, ensuring consistency between the *in vitro* phantom and the Monte Carlo model. A refractive index of 1.37 was assumed for all tissue layers, with the surrounding medium set to air (n=1). The resulting optical coefficients used for each wavelength are summarized in Table 2.

#### 2.2.2 Simulation parameters

The photon-transport simulations were implemented in MATLAB (R2024a) using a custom Monte Carlo photon-tracing algorithm based on the statistical approach of Wang and Jacques.^39^ The model allowed for full control over layer geometry, optical properties, and detectors configuration.

A total of $1\times 10^{7}$ photons were launched per wavelength (530, 665, and 940 nm). Light source was modeled as a normally incident Gaussian beam $1/e^{2}$ radius of 1 mm, representative of the LED emission profile. Each photon was assigned an initial statistical weight of 1 and propagated throughout the multilayered medium. Photon step sizes were randomly sampled from an exponential distribution governed by the total interaction coefficient:

$$\mu_{t}=\mu_{a}+\mu_{s},$$

where the scattering coefficient was derived from the reduced scattering coefficient using,

$$\mu_{s}=\frac{\mu_{s}^{\prime }}{1-g}.$$

Photon scattering directions were determined using the Henyey-Greenstein phase function. Absorption was modeled by decrementing photon weight proportionally to $\mu_{a}/\mu_{t}$ and reflections at tissue interfaces were handled using Fresnel equations. Photons crossing into a new layer were assigned its corresponding optical parameters. Photons with a weight below $10^{-4}$ underwent Russian roulette termination to preserve energy balance.

Two detector configurations were simulated to replicate the experimental setup. Reflectance mode, where photons exiting the top surface within a 3 mm radius circular detector offset 4 mm laterally from the light source were recorded; and transmittance mode, where photons exiting the bottom surface within a 3 mm radius circular detector centered on the illumination axis were detected.

Simulations were performed for the three skin pigmentation layers (pale, medium, and dark) by varying the absorption and scattering coefficients of the skin layers according to the experimentally measured optical properties. All other layers, such as adipose tissue and blood, remained constant across simulations.

Diffuse reflectance (R), transmittance (T), and absorption (A) were computed as the ratio of the total detected photon weight to the number of photons launched, excluding the specular reflection component. Spatial energy deposition was recorded in a fluence density matrix (200×200 bins), from which fluence maps were generated to visualize photon confinement and propagation across wavelengths and skin tones.

Optical path length (OPL) was calculated as the cumulative distance travelled within the tissue by each reflected photon, weighted by its exited energy, representing the effective photon migration distance contributing to the detected signal. Layer-specific absorption fractions were computed to quantify the contribution of each tissue-simulating layer to total absorption. A skin contribution index (SCI) was defined as the sum of the absorption in the top and bottom skin layers, expressed as a percentage of total absorbed energy. Penetration depth was quantified using fluence-based metrics, defined as the depth at which 50% of the cumulative absorbed energy occurred.

### 2.3 Correlation Analysis

To compare Monte Carlo simulations with experimental measurements, the simulated skin layer absorption fraction quantified using the SCI was correlated with experimentally measured PPG AC/DC ratios. In addition, simulated diffuse reflectance and transmittance values were correlated with the corresponding *in vitro* PPG peak-to-peak amplitudes across wavelengths and skin tones. Pearson’s correlation coefficient was used to assess the agreement between simulated optical metrics and experimental PPG signal features.

## 3 Results

The results of this study are presented in three main parts: (i) the evaluation of the fabricated vascular finger phantom and its ability to reproduce physiologically relevant PPG signals across three skin pigmentation levels, (ii) analysis of photon-transport behavior obtained from Monte Carlo simulations, and (iii) the correlation between experimental and simulated results to validate the influence of skin pigmentation on optical PPG performance.

The *in vitro* experiments were conducted to verify that the advanced finger phantom could generate PPG signals in both reflectance and transmittance configurations under pulsatile flow conditions. The Monte Carlo simulations were then used to interpret the optical behavior observed experimentally, specifically, how the different skin layers influence photon transport, penetration depth, absorption, and detected intensity.

### 3.1 *In Vitro* Experiment

**Fig. 7:**
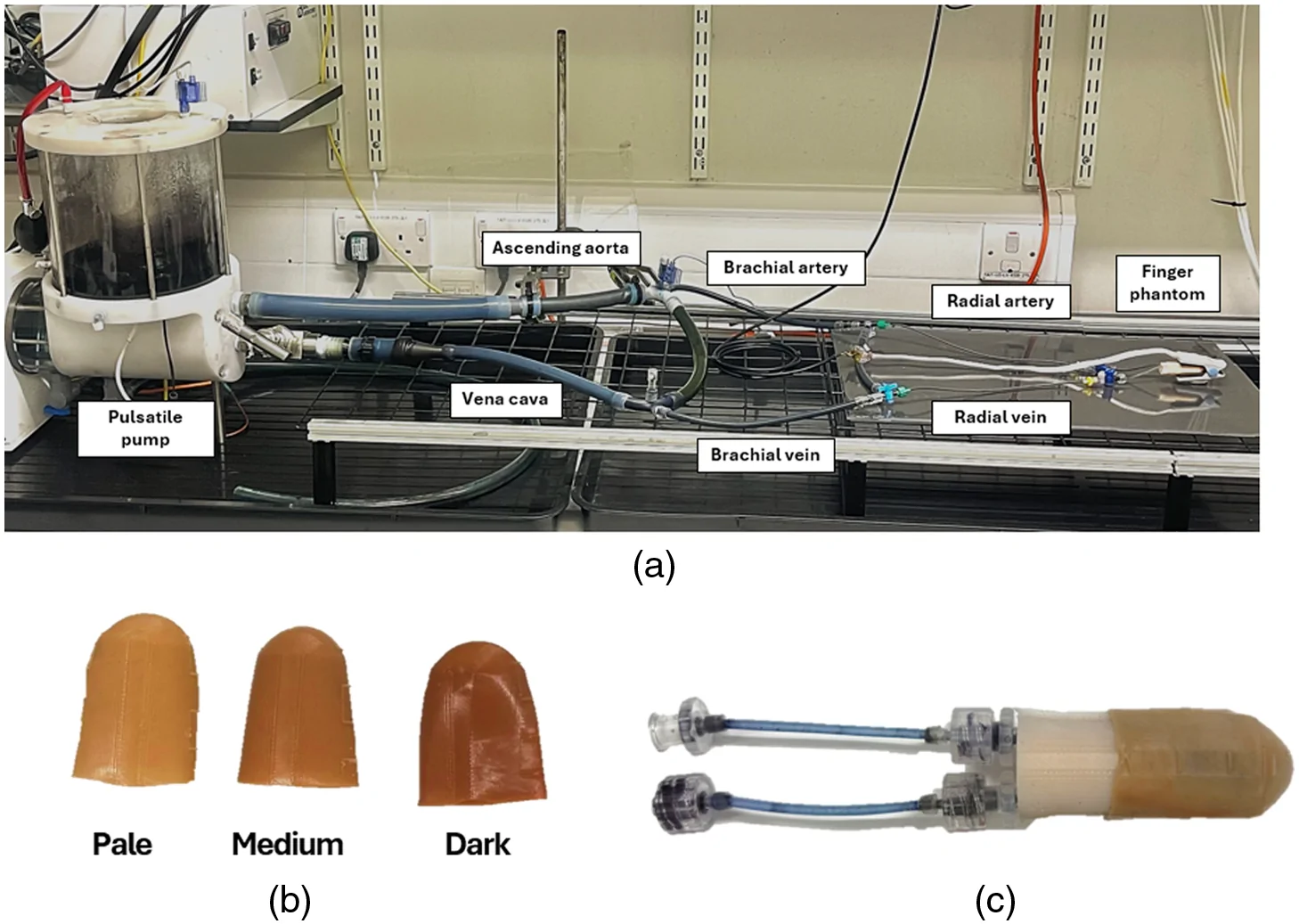
*In vitro* experimental setup. (a) Cardiovascular flow loop used to perfuse the vascular finger phantom under physiologically representative pulsatile flow conditions, incorporating the pulsatile pump, ascending aorta, brachial artery, radial artery, and returning venous pathway. (b) The three interchangeable skin tone layers represents pale, medium, and dark pigmentation. (c) The vascular finger phantom with a skin tone layer.

The fabricated finger phantom enabled continuous fluid perfusion through the three-layer channel vascular network without any leak or occlusion. Pulsatile flow at 60 bpm was successfully generated using a programmable pump, producing pressure variations that resulted in detectable optical modulations at the fingertip. Figure 7 shows the experimental setup used for both reflectance and transmittance configurations, including the cardiovascular flow loop, the three interchangeable skin tone layers representing pale, medium, and dark skin pigmentation, and the finger phantom connected to the circuit.

**Fig. 8:**
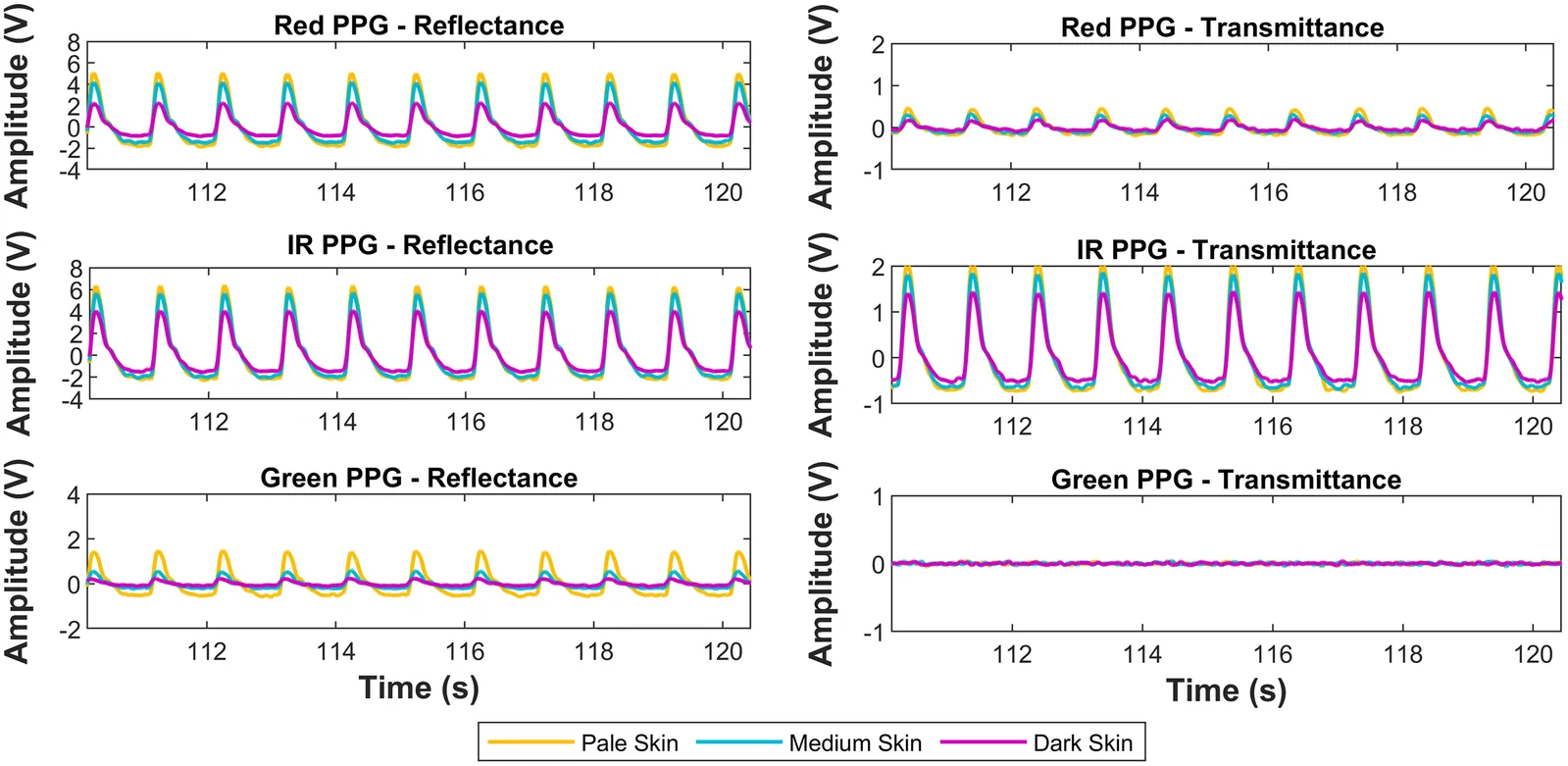
Comparison of reflectance (left) and transmittance (right) PPG signals from the vascular finger phantom across three skin tone conditions: pale (yellow), medium (turquoise), and dark (purple). Each plot shows a 10-s window of the PPG signal for the red (top), infrared (middle), and green (bottom).

PPG signals were successfully recorded for all three skin tones at 530, 665, and 940 nm in both acquisition modes. The waveforms exhibited characteristic physiological PPG signals. Figure 8 presents a 10-s window of the recorded PPG signals for pale, medium, and dark skin tones in reflectance (left) and transmittance mode (right), illustrating differences in signal morphology and confirming the finger phantom’s ability to generate pulsatile signals in both measurement modes.

Quantitative assessment was performed using the peak-to-peak AC amplitude, SNR, and AC/DC ratio, which together characterize PPG signal quality and sensitivity to skin pigmentation. Table 3 summarizes the median and interquartile range (IQR) of these metrics across wavelengths and skin tones.

**Fig. 9:**
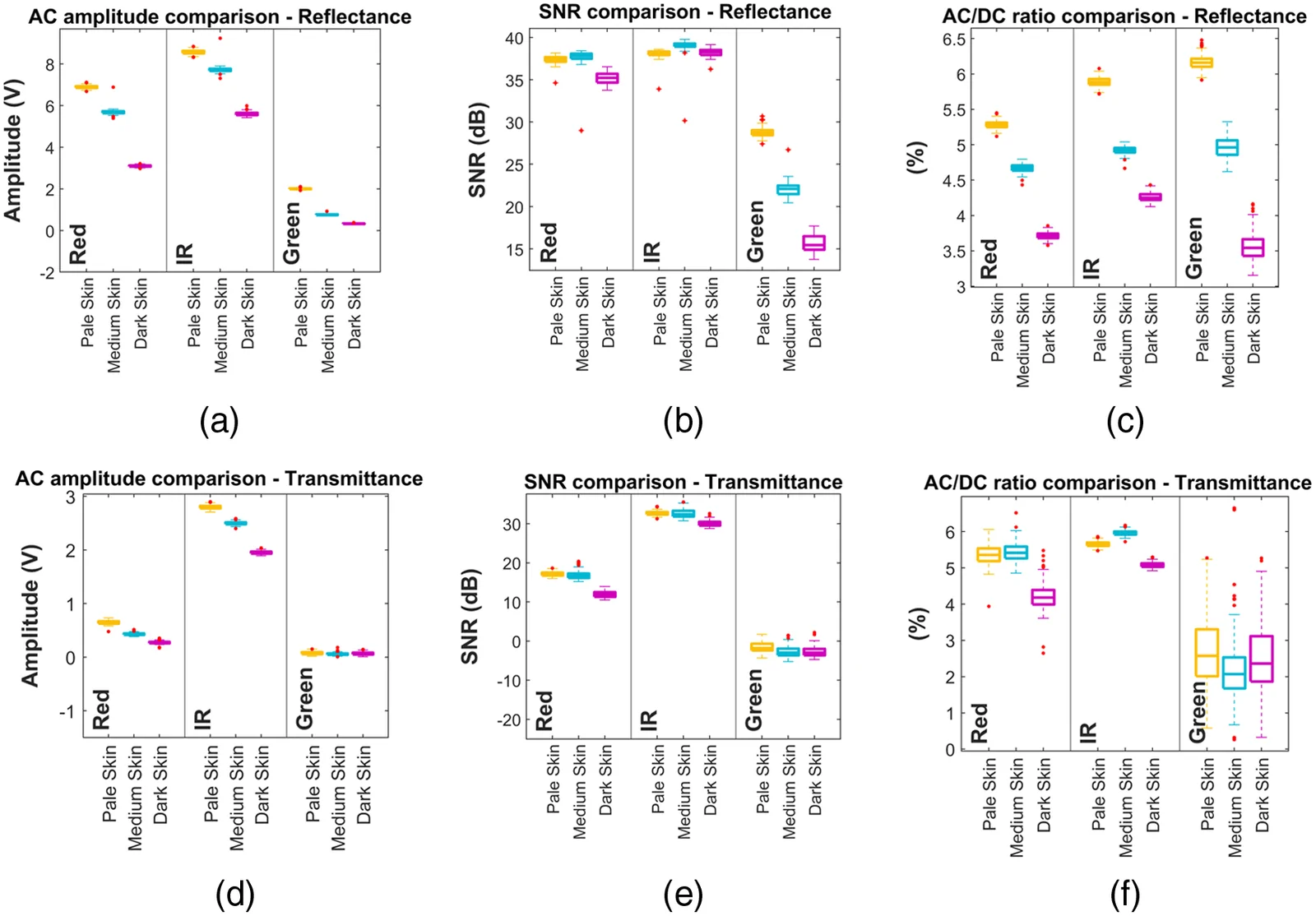
Boxplot comparison for PPG signal quality metrics across skin tones and wavelengths. Peak-to-peak AC amplitude, signal-to-noise ratio (SNR), and AC/DC ratios are shown for pale (yellow), medium (turquoise), and dark (purple) skin layers under reflectance (a)–(c) and transmittance (d)–(f) measurement configurations. Boxes indicate median and interquartile ranges, with red asterisks that denote outliers.

In reflectance mode, clear PPG waveforms were recorded across all wavelengths and skin tones, confirming the finger phantom enabled reliable optical detection of pulsatile blood-volume modulation. As illustrated in Figs. 9(a)–9(c), infrared produced the largest peak-to-peak amplitudes and the most stable signals, followed by red and then green wavelengths. Quantitatively, infrared reflectance amplitudes decreased from a median of 8.57 V for pale skin to 5.59 V for dark skin, while red amplitudes showed a similar attenuation from 6.89 to 3.09 V (Table 3). Green reflectance signals were substantially weaker and exhibited the strongest sensitivity to skin pigmentation, decreasing from 2.01 V in pale skin to 0.33 V in dark skin.

In transmittance mode, the observed trends differed [Figs. 9(d)–9(f)]. As expected, the green wavelength produced negligible signals across all skin tones, reflecting its shallow penetration depth and predominant absorption in superficial layers, preventing light from reaching the deeper vascular regions. In contrast, red and infrared wavelengths, which were the target wavelengths for transmittance measurements, produced detectable PPG signals. Their amplitudes were lower than those in reflectance mode, reflecting the longer optical path through the phantom. Infrared exhibited the highest transmittance signal amplitude, with median amplitudes decreasing from 2.80 V for pale skin to 1.95 V for dark skin, while red amplitudes decreased from 0.65 to 0.27 V (Table 3). Increasing skin pigmentation resulted in a consistent reduction in transmittance signal amplitude, reflecting higher absorption within the dark pigmented skin layers.

**Table 3:** Median and interquartile ranges (IQR) for peak-to-peak amplitude and signal-to-noise ratio (SNR) for red, infrared (IR), and green wavelengths, by skin tone condition in reflectance and transmittance photoplethysmography (PPG) modes. Corresponding sample sizes (n) are also reported for each feature; the difference in sample sizes between metrics arises because AC amplitude and AC/DC are derived from individual pulse cycles, whereas SNR is estimated from fixed time windows.

		Pale skin		Medium skin		Dark skin	
Skin layer		Median	IQR	Median	IQR	Median	IQR
AC Amplitude (V) (n=298)							
Reflectance	Red	6.891	0.081	5.687	0.089	3.094	0.052
	IR	8.567	0.116	7.712	0.101	5.588	0.105
	Green	2.005	0.037	0.763	0.031	0.330	0.022
Transmittance	Red	0.647	0.043	0.428	0.026	0.271	0.026
	IR	2.795	0.045	2.495	0.034	1.951	0.036
	Green	0.074	0.037	0.056	0.023	0.063	0.034
SNR (dB) (n=60)							
Reflectance	Red	37.410	0.540	37.818	0.592	35.217	1.039
	IR	38.081	0.417	39.114	0.367	38.267	0.585
	Green	28.708	0.599	22.083	1.009	15.437	1.593
Transmittance	Red	17.123	0.711	16.581	1.136	11.980	1.193
	IR	32.570	0.626	32.387	1.448	29.941	0.935
	Green	−1.799	1.704	−2.948	1.696	−2.960	1.625
AC/DC (%) (n=297)							
Reflectance	Red	5.280	0.062	4.673	0.073	3.706	0.062
	IR	5.887	0.081	4.920	0.064	4.252	0.080
	Green	6.160	0.113	4.962	0.203	3.542	0.234
Transmittance	Red	5.358	0.350	5.415	0.325	4.181	0.399
	IR	5.648	0.090	5.956	0.082	5.078	0.095
	Green	2.575	1.294	2.070	0.856	2.361	1.252

Signal quality trends were further supported by the SNR and AC/DC ratio metrics (Fig. 9 and Table 3). In reflectance mode infrared achieved the highest SNR values across all skin tones (∼38 to 39 dB), followed by red, while green exhibited a marked decrease in SNR with increasing pigmentation, with ∼28  dB in pale skin and falling to ∼15  dB in dark skin. In transmittance mode, SNR values were lower overall due to the increased optical path length, with infrared remaining the most robust wavelength (∼33  dB in pale skin, decreasing to ∼30  dB in dark skin), followed by red (∼17  dB in pale skin, decreasing to ∼12  dB in dark skin). Green transmittance signals exhibited no pulsatile content across all skin tones.

The AC/DC ratio exhibited similar behavior for the wavelengths and skin pigmentation effects. In reflectance mode, AC/DC ratios decreased progressively with increasing skin pigmentation for all wavelengths, with the largest reductions observed for green light. In transmittance mode, infrared and red wavelengths maintained comparable AC/DC ratios across skin tones, while green showed increased variability, consistent with its limited penetration depth.

Collectively, these results demonstrate that the improved perfusion phantom produces physiologically realistic PPG waveforms across multiple skin tones and wavelengths for reflectance and transmittance mode.

### 3.2 Monte Carlo Simulation Results

Simulations incorporating pale, medium, and dark skin tones were performed using the experimentally derived optical properties of the fabricated phantom materials (Table 2). By varying only the optical properties of the skin layer while keeping tissue geometry and adipose and blood optical properties constant, the simulations isolate the effect of skin pigmentation.

Simulations were conducted at 530, 665, and 940 nm under identical geometry and illumination conditions, enabling direct comparison of photon transport and energy deposition metrics across wavelengths and skin tones.

#### 3.2.1 Photon transport and energy deposition behavior

**Fig. 10:**
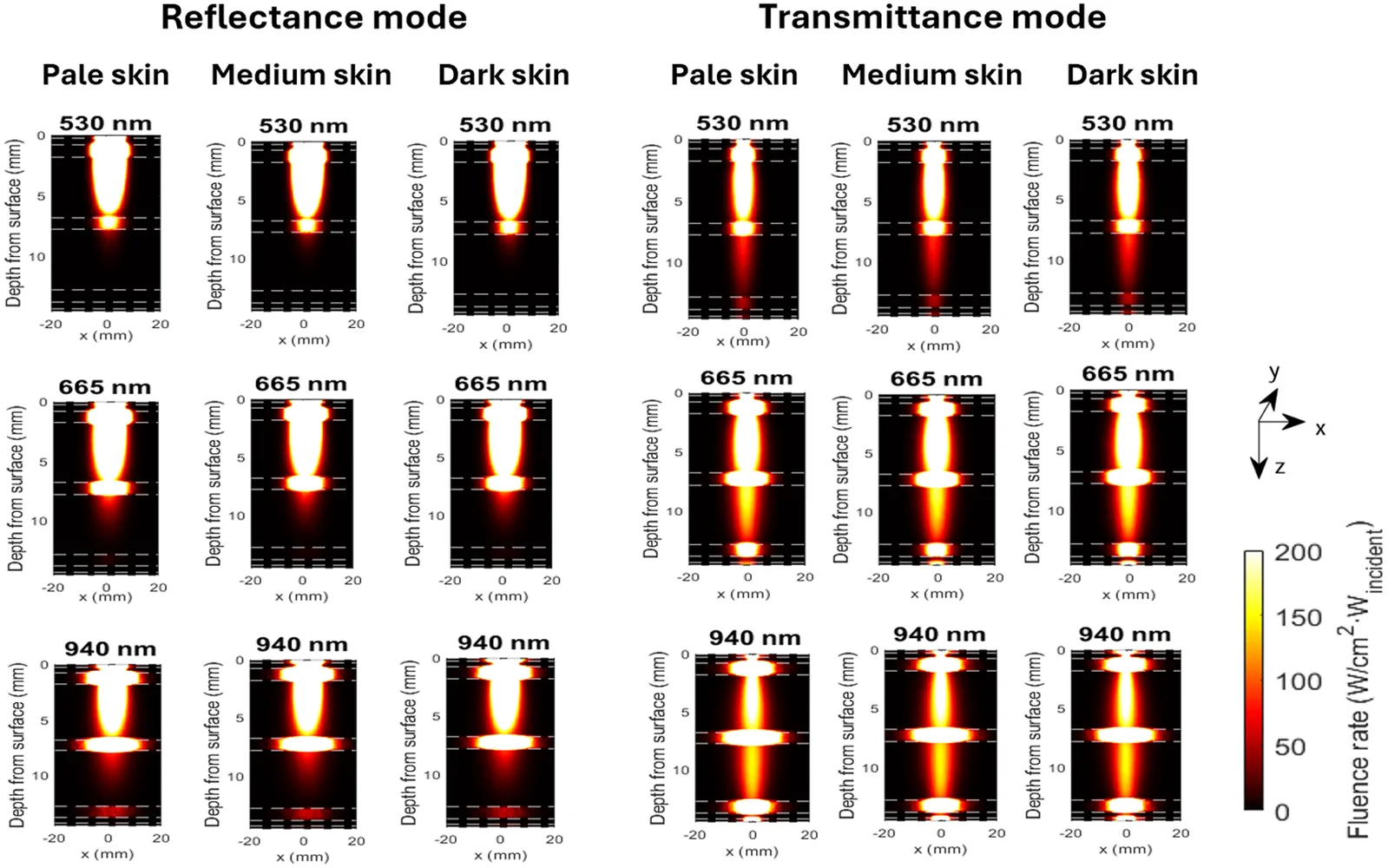
Simulated photon fluence distributions for pale, medium, and dark skin tones at 530, 655, and 940 nm in reflectance (left) and transmittance (right) modes.

Figure 10 shows the simulated photon fluence distributions for pale, medium, and dark skin tones at 530, 665, and 940 nm in reflectance and transmittance modes. The tissue depth (z-axis) and lateral distance (x-axis) correspond to the simulated tissue region, with dashed lines indicating the interfaces between layers. In reflectance, photon energy was concentrated near illumination site, with shallow interaction at 530 nm, extending slightly deeper at 665 nm, and reaching the middle blood layer at 940 nm. In transmittance, green light was fully attenuated within superficial layers, while limited transmission was observed at 665 nm, and pronounced propagation through the full tissue depth occurred at 940 nm.

Across all wavelengths, increasing skin pigmentation reduced the overall fluence magnitude due to higher skin absorption. Although these differences are visually subtle, they are consistent with the expected attenuation behavior and support the wavelength-dependent transition from shallow to deep optical interaction within the finger phantom model.

**Fig. 11:**
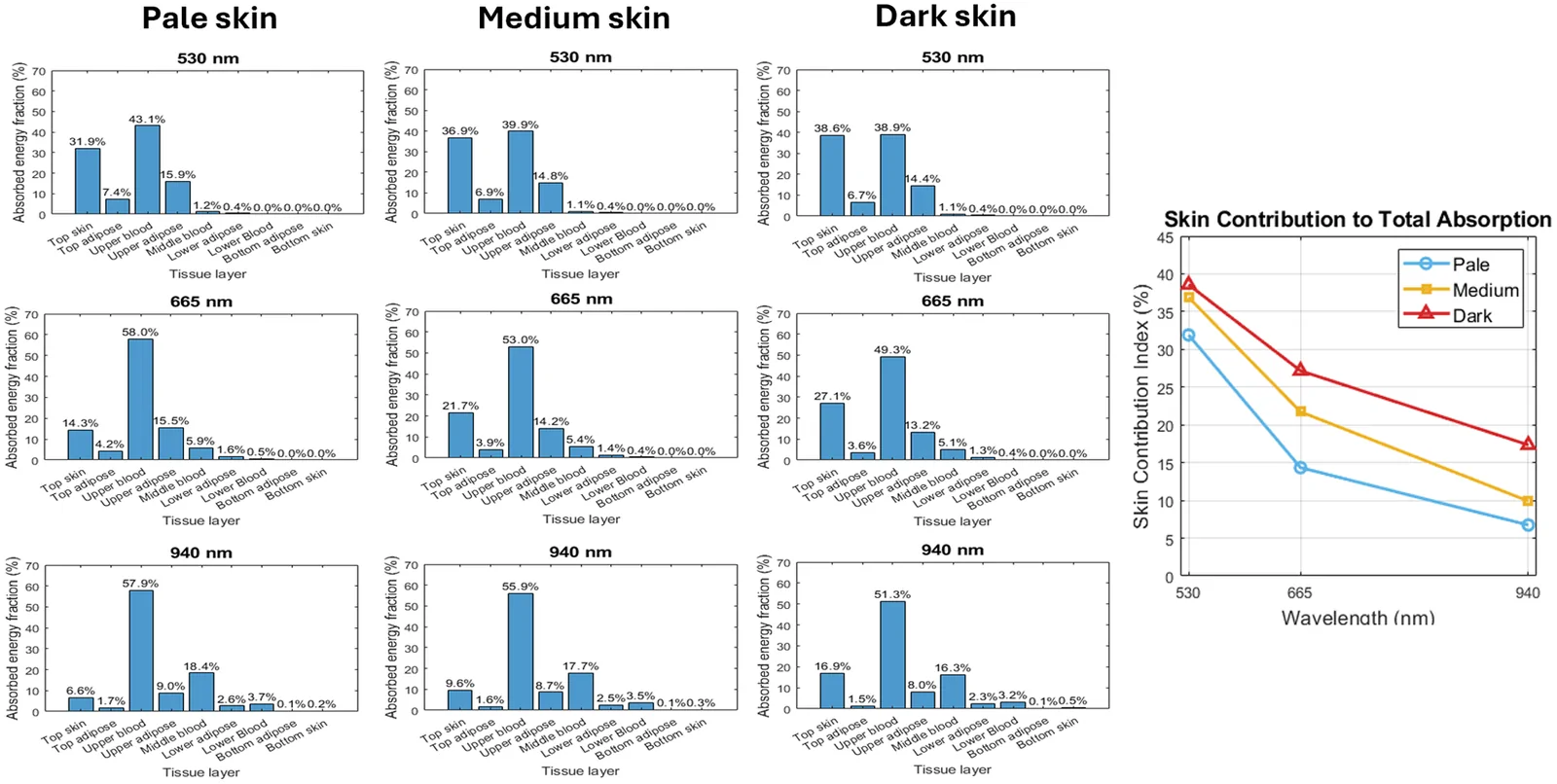
Wavelength and pigmentation-dependent absorption behavior obtained from Monte Carlo simulations. The bar plots show the layer-specific absorbed energy fractions for pale, medium, and dark skin tones at 530, 665, and 940 nm. The line plot (right) quantifies the Skin Contribution Index (SCI), defined as the percentage of total absorbed energy within the pale (blue), medium (yellow), and dark (red) skin tones.

To quantify this behavior, Fig. 11 summarizes the distribution of absorbed photon energy across tissue layers and the resulting skin contribution index (SCI). At 530 nm, absorption was dominated by the skin and upper blood layers, together accounting for more than 70% of total absorbed energy. At 665 nm, absorption extended further into the adipose and middle blood layer regions, while at 940 nm, absorption became more evenly distributed throughout the tissue depth, reflecting enhanced photon penetration.

The SCI decreased with increasing wavelength and increased with skin pigmentation, confirming that darker skin retains a larger fraction of absorbed energy within the skin layers across all wavelengths. Dark skin exhibited the highest SCI values, reaching nearly 40% at 530 nm compared with lower contributions for medium and pale skin tones, 37% and 32%, respectively.

#### 3.2.2 Energy balance and photon transport metrics

**Fig. 12:**
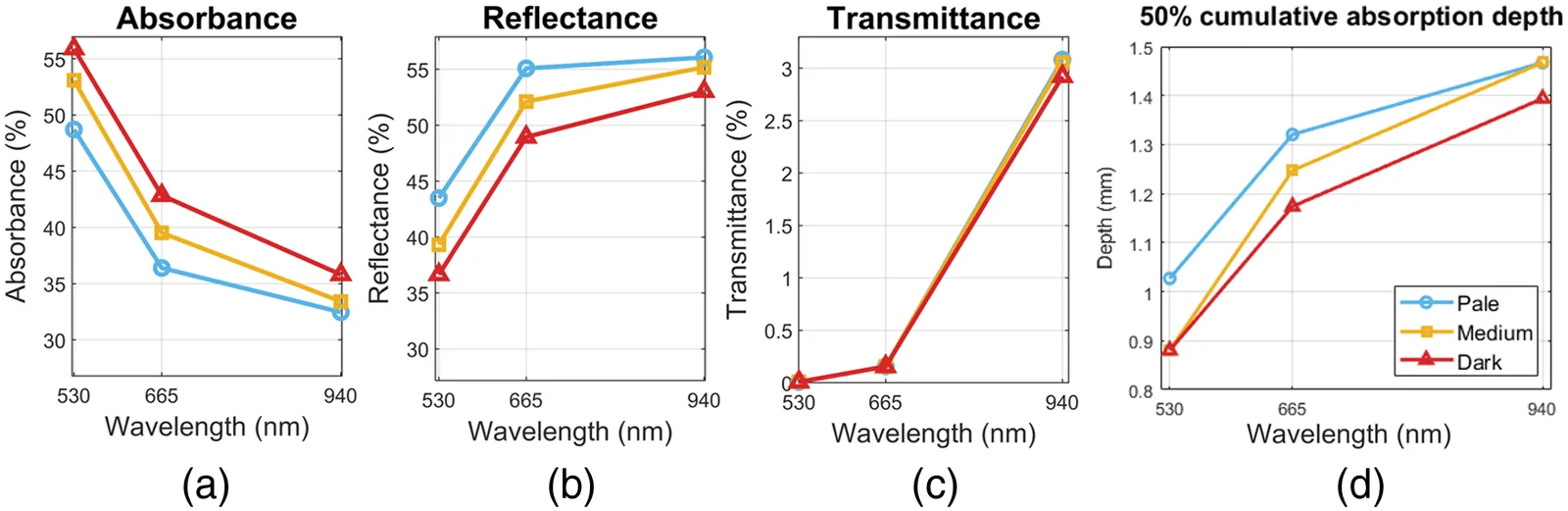
Monte Carlo-derived photon transport metrics across wavelengths (530, 665, and 940 nm) for pale (blue), medium (yellow), and dark (red) skin tones: (a) absorbed energy fraction, (b) diffuse reflectance, (c) transmittance, and (d) fluence-based 50% cumulative absorption depth ($Z_{50}$).

Figure 12 summarizes the simulated photon energy balance for pale, medium, and dark skin tones across all wavelengths. Absorption dominated the photon energy balance, decreasing progressively as wavelength increased, from ∼56% at 530 nm to 32% at 940 nm for the dark skin. Reflectance increased slightly with wavelength, indicating deeper photon penetration and reduced superficial attenuation. Transmittance remained negligible at 530 and 665 nm and increased slightly at 940 nm.

To characterize photon penetration, fluence-based photon penetration depth was quantified using the 50% cumulative absorption depth. This metric represents the depth at which most photon energy is deposited within the tissue layers. The results show that photon penetration increased with wavelength. At 530 nm, half of the total absorbed energy was confined within the upper 1 mm, extending to around 1.3 mm at 665 nm and 1.5 mm at 940 nm. Across all wavelengths, darker pigmentation produced slightly shallower penetration depths due to the increased absorption, especially in the visible range.

### 3.3 Correlation between Experimental and Simulated Data

**Fig. 13:**
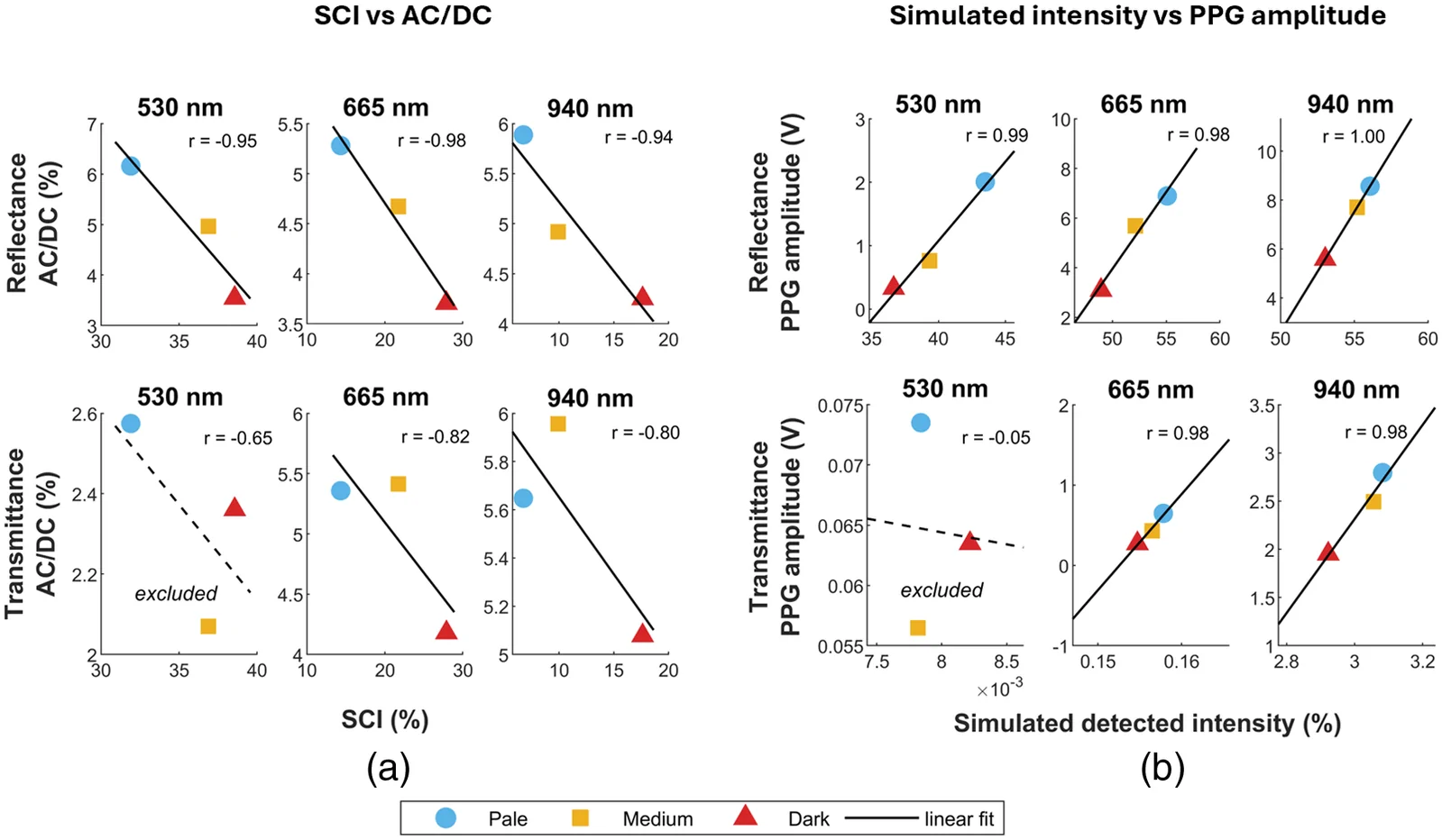
Correlation between Monte Carlo simulations and *in vitro* PPG measurements. (a) Relationship between Skin Contribution Index (SCI) and AC/DC ratio for reflectance and transmittance modes across wavelengths. (b) Relationship between Monte Carlo simulated detected intensity and experimentally measured PPG peak-to-peak amplitude. Data points represent pale, medium, and dark skin tone layers of the finger phantom, solid lines indicate linear fits, and Pearson’s r-values are reported. The 530 nm transmittance results are shown for completeness but excluded from quantitative interpretation due to insufficient signal, given the shallow penetration depth of this wavelength.

Strong agreement was observed between Monte Carlo-simulated optical trends and experimentally measured PPG metrics across wavelengths and skin tones. In reflectance mode, AC/DC ratios decreased consistently with increasing skin pigmentation at all wavelengths, yielding strong negative correlations (|r|≥0.94). In transmittance mode, negative correlations were observed at 665 and 940 nm (|r|≈0.8), while no reliable PPG signal was detected at 530 nm due to the shallow penetration depth of green light, and this wavelength was excluded from quantitative interpretation [Fig. 13(a)].

Simulated detected intensity showed strong positive agreement with experimentally measured PPG peak-to-peak amplitude. In reflectance mode, correlations were consistently high across all wavelengths (r≥0.98), indicating that pigmentation-driven reductions in simulated diffuse reflectance translated directly into reduced signal amplitude. In transmittance mode, similarly strong agreement was observed at 665 and 940 nm (r≈0.98), whereas the 530 nm transmittance mode was excluded due to insufficient signal presence [Fig. 13(b)].

Overall, the correlation analysis demonstrates that Monte Carlo simulated changes in photon transport with increasing skin pigmentation are closely reflected in both PPG amplitude and AC/DC ratio, with the strongest and most consistent agreement observed in reflectance mode. Although the correlation analysis is descriptive given the three data points representing the three skin tone conditions, the consistent direction and magnitude of correlations across wavelengths and measurement modes suggest that the observed relationships between simulated photon transport and experimental PPG metrics are robust and representative of the underlying optical behavior.

## 4 Discussion

This study combined an advanced vascular finger phantom with Monte Carlo modelling to investigate how skin pigmentation alters PPG signals in both reflectance and transmittance measurement modes. Building on prior phantom work that demonstrated a decrease in signal quality with increasing skin pigmentation in reflectance measurements,^12^ the present design extends the experimental capability to incorporate transmittance mode PPG signals and directly links observed signal changes to simulated photon transport and absorption behavior.

Although pulse oximetry applications have highlighted the potential impact of skin pigmentation in optical measurements, the present study focuses specifically on the underlying PPG signal quality and its dependence on light–tissue interaction. To achieve this, fixed acquisition conditions were employed to isolate the intrinsic optical effects of pigmentation, enabling controlled comparison of wavelength-dependent attenuation and signal quality metrics without confounding effects from adaptive system responses. This approach aims to provide insight into the fundamental mechanisms governing PPG signal formation. This is particularly relevant for emerging wearable technologies that use algorithms based on PPG waveform morphology and derived features, such as blood pressure estimation,^40^^,^^41^ arterial stiffness assessment,^42^^–^^44^ and other cardiovascular markers, where reduced signal quality and increased variability may directly impact algorithm performance.

The multilayer vascular architecture enables a more physiologically representative interaction between light and pulsatile blood volume by incorporating both superficial and deeper vascular layers. Unlike the previous single-vessel phantom, the present design incorporates three distributed perfusion layers spanning superficial and deeper tissue regions, allowing evaluation of wavelength-dependent photon penetration within a more physiologically representative vascular environment. Importantly, the added vascular complexity did not change the expected optical behavior that melanin-simulating absorbers attenuate shorter wavelengths more strongly, as this could also be demonstrated using simpler single-vessel models.^8^^,^^10^ The purpose of the present vascular network was instead to address a limitation of such simplified models: their restricted vascular interaction volume and non-physiological depth distribution of pulsatile blood. This distributed vascular structure increases light-blood interaction across tissue depths, enabling stable acquisition of PPG signals across reflectance and transmittance modes and allowing more consistent comparison of signal quality metrics across wavelengths and skin tones. This includes conditions where signal generation was particularly challenging in our previous study,^12^ such as shorter wavelengths under dark skin pigmentation, where in vivo signals are still typically observable. This was made possible by the 3D-printed PVA sacrificial template fabrication method, which enables the formation of small, compliant, geometrically complex channel networks that would be difficult to achievable through conventional single-vessel casting approaches. The resulting distributed vascular network more closely reflects the spatial organization of physiological microvascular perfusion within the highly vascularized fingertip, representing a fundamental fabrication advance over prior simplified phantom designs. Therefore, this specific advancement is not the demonstration that melanin absorption is wavelength dependent, which is already well established, but the ability to produce measurable PPG signals within a more physiologically representative depth-distributed vascular environment and to evaluate how distributed pulsatile blood volume interacts with wavelength-dependent photon penetration in both reflectance and transmittance geometries, which could not be reliably achieved using the previous single-vessel configuration.

Across the *in vitro* measurements, increasing pigmentation produced a consistent reduction in PPG strength, which is expected as higher skin absorption reduces the light available to interrogate pulsatile blood volume and reach the detector, as demonstrated in prior studies.^7^^,^^45^ This effect was most apparent at shorter wavelengths and in reflectance mode, where the detected photons are most influenced by superficial losses, whereas longer wavelengths better preserved pulsatility due to deeper tissue interaction.^46^ The integrated simulations provide a direct physical insight into the underlying photon transport mechanisms responsible for these trends. By linking experimental observations with photon-based metrics, this work extends previous phantom studies beyond qualitative signal comparison.

Monte Carlo simulations provided a mechanistic explanation for the experimentally observed signal attenuation with increasing skin pigmentation. Fluence distributions demonstrated that higher skin absorption progressively reduced photon energy reaching deeper vascular layers, particularly at shorter wavelengths. This behavior was quantified through the skin contribution index (SCI), which increased with pigmentation and decreased with wavelength. These findings are consistent with prior modeling studies of light–tissue interaction in PPG, which show that melanin-dominated absorption redistributes photon energy toward superficial layers, thereby limiting sensitivity to pulsatile blood volume changes.^17^^,^^47^

Similar trends have also been reported in Monte Carlo studies of pulse oximetry, where increasing melanin concentration leads to a reduction in AC/DC amplitude and a systematic drift in the ratio-of-ratios, driven by wavelength-dependent attenuation of the pulsatile signal.^48^ In particular, these studies demonstrate that higher melanin levels reduce the detected pulsatile component and alter the relative contribution of red and infrared wavelengths, contributing to a bias in ${\mathrm{SpO}}_{2}$ estimation. The present results are in agreement with these observations, as the experimentally measured reductions in PPG amplitude, SNR, and AC/DC ratio with increasing pigmentation reflect the same underlying mechanism of reduced photon penetration and diminished interaction with pulsatile blood volume.

The 50% cumulative absorption depth increased with wavelength, indicating progressively deeper photon interaction at red and infrared wavelengths. Across all wavelengths, darker pigmentation resulted in slightly shallower penetration depths, reflecting increased superficial absorption. Energy balance analysis showed that absorption was the dominant mechanism of photon fate at all wavelengths and pigmentation conditions. Together, these results demonstrate that pigmentation primarily affects PPG signal formation by altering where photon energy is deposited within the tissue.

Strong correlation between Monte Carlo predictions and experimental PPG metrics validates the combined modeling-phantom approach. Simulated diffuse reflectance and transmittance closely matched measured PPG amplitudes, while SCI showed strong agreement with AC/DC ratios, particularly in reflectance mode. Although the correlation analysis is descriptive in nature, reflecting the three discrete and physically distinct skin pigmentation levels from the experimental design, the consistent directional agreement between Monte Carlo predictions and experimental PPG metrics across multiple wavelengths and measurement modes supports the validity of the combined modelling-phantom approach. Notably, cases where a correlation was not expected, such as the 530 nm transmittance condition where signal penetration is limited, did not exhibit meaningful alignment, further indicating that the reported Pearson correlations reflect genuine optical behavior rather than coincidental agreement. These findings demonstrate that skin pigmentation-driven changes in photon transport are directly reflected in measured PPG features, strengthening the confidence that the observed attenuation trends arise from optical mechanisms rather than artifacts of phantom design or experimental variability. Such agreement is rarely demonstrated in phantom studies and highlights the value of integrating modeling alongside in vitro experimentation.

One limitation of this study is the simplified blood optical properties, as the blood-mimicking fluid exhibits a monotonic absorption spectrum, rather than the wavelength-dependent hemoglobin absorption spectra observed *in vivo*,^49^ which vary with oxygenation level, and does not replicate the high scattering coefficient of red blood cells. However, this blood-mimicking fluid preserves the expected trend of higher absorption at shorter wavelengths and lower absorption in the infrared region, allowing relative comparisons of signal behavior. Similar nigrosine-based blood-mimicking fluids have been used in optical phantom studies to investigate light–tissue interactions in *in vitro* systems.^8^^,^^10^ Regarding scattering, while red blood cells contribute substantially to blood scattering *in vivo*, this effect predominantly governs photon interaction within the vascular compartment; consequently, increased blood scattering would not be expected to alter the directional relationship between skin pigmentation and PPG signal attenuation reported in this study. Nevertheless, incorporating physiologically representative blood optical properties, including wavelength-dependent hemoglobin absorption and realistic red blood cells scattering, remains an important goal for future phantom improvements, particularly for extending this framework toward pulse oximetry applications.

A second limitation relates to the use of discrete wavelengths in the Monte Carlo simulations, whereas the experimental PPG system employs LED sources with finite spectral bandwidths. In LED-based PPG systems, the measured signal represents the convolution of the LED emission spectrum, tissue optical properties, and detector sensitivity, which may lead to a spectral shift in darker skin tones, particularly in the red spectral region.^50^ As a result, the simulations are more representative of narrowband illumination and do not fully capture the spectral response of the experimental setup.

A third limitation is that the scattering properties of the skin and adipose tissue layers do not fully reproduce the wavelength-dependent decrease in scattering typically observed in biological tissue. The greatest divergence from reported literature values was observed at 530 nm, where ${\mathrm{TiO}}_{2}$ particle scattering characteristics differ from the scattering behavior of biological tissue constituents such as collagen fibers and cell organelles. At this wavelength, an underestimation of scattering would reduce photon pathlength in the superficial layer, potentially leading to an underestimation of the filtering effect of skin pigmentation on the detected signal. However, as melanin absorption rather than scattering is the dominant driver of superficial attenuation at 530 nm, the directional trends in skin pigmentation-driven signal attenuation reported here are not expected to be fundamentally altered. Future work will focus on incorporating wavelength-dependent scattering behavior and physiologically representative oxygenated and deoxygenated blood levels to further improve optical realism and extend the framework.

Overall, this study contributes to the phantom-based literature^8^^,^^10^^,^^11^^,^^13^^,^^51^^,^^52^ by providing a quantitative and mechanistic assessment of how skin pigmentation influences PPG signal quality. Although previous phantom studies have incorporated varying skin tone layers, the present work extends this approach by evaluating specific PPG features, including peak-to-peak amplitude, SNR, and AC/DC ratio, under controlled and physiologically relevant perfusion conditions. By integrating the advanced vascular phantom with Monte Carlo modelling, this study offers a robust framework to isolate the optical effects of skin pigmentation on PPG quality.

## 5 Conclusion

This study investigated the impact of skin pigmentation on photoplethysmography using an advanced perfused vascular finger phantom combined with Monte Carlo modelling. The 3D-printed PVA sacrificial template approach enabled the fabrication of a geometrically complex, distributed vascular network that represented a fundamental advance over simplified single-vessel phantom designs, allowing PPG signal acquisition across all wavelengths and skin tones. Increasing pigmentation produced consistent reductions in PPG amplitude, SNR, and AC/DC ratio across wavelengths, consistent with increased absorption in the skin layers. These effects were most pronounced at shorter wavelengths and in reflectance mode, where superficial absorption dominates photon transport, while longer wavelengths preserved deeper vascular sensitivity. Monte Carlo simulations provided mechanistic insights by linking these trends to superficial photon energy deposition, effective penetration depth, and reduced interaction with pulsatile blood volumes. Consistent directional agreement between simulated optical metrics and experimental PPG features supports the validity of the combined experimental-computational framework. Overall, this combined approach supports improved understanding and evaluation of light–tissue interaction under varying skin pigmentation conditions, with direct relevance to emerging wearable devices and PPG-based applications that extend beyond pulse oximetry, including blood pressure estimation, arterial stiffness assessment, and other cardiovascular markers, where reduced signal quality due to skin pigmentation may directly impact algorithm performance.

## Data Availability

Data produced in this study are available upon reasonable request.

## References

[1] Saunders N. A. Powles A. C. P. Rebuck A. S., “Ear oximetry: accuracy and practicability in the assessment of arterial oxygenation,” Am. Rev. Respirat. Dis. 113(6), 745–749 (1976). ARDSBL 0003-0805 10.1164/arrd.1976.113.6.745 937815

[r2] Merrick E. B. Hayes T. J. C., “Non-invasive measurements of arterial blood oxygen levels,” Hewlett-Packard J. 28, 2–9 (1976). HPJOAX 0018-1153

[r3] Ries A. L. Prewitt L. M. Johnson J. J., “Skin color and ear oximetry,” Chest 96, 287–290 (1989). CHETBF 0012-3692 10.1378/chest.96.2.287 2752811

[r4] Jubran A. Tobin M. J., “Reliability of pulse oximetry in titrating supplemental oxygen therapy in ventilator-dependent patients,” Chest 97, 1420–1425 (1990). CHETBF 0012-3692 10.1378/chest.97.6.1420 2347228

[5] Cahan C. et al., “Agreement between noninvasive oximetric values for oxygen saturation,” Chest 97, 814–819 (1990). CHETBF 0012-3692 10.1378/chest.97.4.814 2323252

[6] Sjoding M. W. et al., “Racial bias in pulse oximetry measurement,” N. Engl. J. Med. 383, 2477–2478 (2020). 10.1056/NEJMc2029240 33326721 PMC7808260

[7] Setchfield K. et al., “Effect of skin color on optical properties and the implications for medical optical technologies: a review,” J. Biomed. Opt. 29, 010901 (2024). JBOPFO 1083-3668 10.1117/1.JBO.29.1.010901 38269083 PMC10807857

[8] Rodriguez A. J. et al., “Tissue mimicking materials and finger phantom design for pulse oximetry,” Biomed. Opt. Express 15, 2308 (2024). BOEICL 2156-7085 10.1364/BOE.518967 38633081 PMC11019708

[r9] Chen A. I. et al., “Multilayered tissue mimicking skin and vessel phantoms with tunable mechanical, optical, and acoustic properties,” Med. Phys. 43, 3117–3131 (2016). MPHYA6 0094-2405 10.1118/1.4951729 27277058 PMC4884191

[r10] Bhusal A. et al., “Development and characterization of silicone-based tissue phantoms for pulse oximeter performance testing,” J. Biomed. Opt. 29, S33314 (2025). JBOPFO 1083-3668 10.1117/1.JBO.29.S3.S33314 PMC11706025 39776836

[r11] Jenne S. Zappe H., “Multiwavelength tissue-mimicking phantoms with tunable vessel pulsation,” J. Biomed. Opt. 28, 045003 (2023). JBOPFO 1083-3668 10.1117/1.JBO.28.4.045003 37077500 PMC10109273

[r12] Osorio-Sanchez L. May J. M. Kyriacou P., “Evaluation of skin pigmentation effect on photoplethysmography signals using a vascular finger phantom with tunable optical and mechanical properties,” J. Biomed. Opt. 30, 117002 (2025). JBOPFO 1083-3668 10.1117/1.JBO.30.11.117002 41306936 PMC12646468

[13] Afshari A. et al., “Evaluation of the robustness of cerebral oximetry to variations in skin pigmentation using a tissue-simulating phantom,” Biomed. Opt. Express 13, 2909 (2022). BOEICL 2156-7085 10.1364/BOE.454020 35774336 PMC9203096

[14] Chatterjee S. Kyriacou P. A., “Monte Carlo analysis of optical interactions in reflectance and transmittance finger photoplethysmography,” Sensors 19, 789 (2018). SNSRES 0746-9462 10.3390/s19040789 PMC6412556 30769957

[r15] Al-Halawani R. Qassem M. Kyriacou P. A., “Monte Carlo simulation of the effect of melanin concentration on light–tissue interactions for transmittance pulse oximetry measurement,” J. Biomed. Opt. 29, S33305 (2024). JBOPFO 1083-3668 10.1117/1.JBO.29.S3.S33305 39139814 PMC11321364

[r16] Al-Halawani R. Qassem M. Kyriacou P. A., “Monte Carlo simulation of the effect of melanin concentration on light–tissue interactions in reflectance pulse oximetry,” Sensors 25, 559 (2025). SNSRES 0746-9462 10.3390/s25020559 39860931 PMC11769186

[17] Ajmal B.-A. et al., “Monte Carlo analysis of optical heart rate sensors in commercial wearables: the effect of skin tone and obesity on the photoplethysmography (PPG) signal,” Biomed. Opt. Express 12, 7445–7457 (2021). BOEICL 2156-7085 10.1364/BOE.439893 35003845 PMC8713672

[18] Shores J. T., “Anatomy and physiology of the fingertip,” in Fingertip Injuries, pp. 1–9, Springer International Publishing, Cham (2015).

[r19] Harenberg P. S. et al., “Reconstruction of the thumb tip using palmar neurovascular flaps,” Oper. Orthop. Traumatol. 24, 116–121 (2012). 10.1007/s00064-011-0081-3 22430376

[20] Jasionyte G. et al., “The promising role of a superb microvascular imaging technique in the evaluation of raynaud’s syndrome in systemic sclerosis: theory and practical challenges,” Diagnostics 11, 1743 (2021). 10.3390/diagnostics11101743 34679441 PMC8535079

[21] Tilley A. R., The Measure of Man and Woman: Human Factors in Design, Wiley, New York (2002).

[22] Lundström R. et al., “Vibrotactile and thermal perception and its relation to finger skin thickness,” Clin. Neurophysiol. Pract. 3, 33–39 (2018). 10.1016/j.cnp.2018.01.001 30215005 PMC6133778

[r23] Peña A. et al., “Non-invasive optical method for epidermal thickness estimation,” Online J. Biol. Sci. 14, 163–166 (2014). 10.3844/ojbsci.2014.163.166

[r24] Fruhstorfer H. et al., “Thickness of the stratum corneum of the volar fingertips,” Clin. Anat. 13, 429–433 (2000). 10.1002/1098-2353(2000)13:6<429::AID-CA6>3.0.CO;2-5 11111894

[r25] Lintzeri D. A. et al., “Epidermal thickness in healthy humans: a systematic review and meta-analysis,” J. Eur. Acad. Dermatol. Venereol. 36, 1191–1200 (2022). 10.1111/jdv.18123 35366353

[26] Lin Y. et al., “A measurement of epidermal thickness of fingertip skin from OCT images using convolutional neural network,” J. Innov. Opt. Health Sci. 14, 1–7 (2021). 10.1142/S1793545821400058

[27] Shimojo Y. et al., “Measurement of absorption and reduced scattering coefficients in Asian human epidermis, dermis, and subcutaneous fat tissues in the 400- to 1100-nm wavelength range for optical penetration depth and energy deposition analysis,” J. Biomed. Opt. 25, 045002 (2020). JBOPFO 1083-3668 10.1117/1.JBO.25.4.045002 32356424 PMC7191311

[r28] Salomatina E. et al., “Optical properties of normal and cancerous human skin in the visible and near-infrared spectral range,” J. Biomed. Opt. 11, 064026 (2006). JBOPFO 1083-3668 10.1117/1.2398928 17212549

[r29] Bashkatov A. N. et al., “Optical properties of human skin, subcutaneous and mucous tissues in the wavelength range from 400 to 2000 nm,” J. Phys. D Appl. Phys. 38, 2543–2555 (2005). 10.1088/0022-3727/38/15/004

[r30] Simpson C. R. et al., “Near-infrared optical properties of ex vivo human skin and subcutaneous tissues measured using the Monte Carlo inversion technique,” Phys. Med. Biol. 43, 2465–2478 (1998). PHMBA7 0031-9155 10.1088/0031-9155/43/9/003 9755939

[31] Peters V. G. et al., “Optical properties of normal and diseased human breast tissues in the visible and near infrared,” Phys. Med. Biol. 35, 1317–1334 (1990). PHMBA7 0031-9155 10.1088/0031-9155/35/9/010 2236211

[32] Saager R. B. et al., “In vivo measurements of cutaneous melanin across spatial scales: using multiphoton microscopy and spatial frequency domain spectroscopy,” J. Biomed. Opt. 20, 066005 (2015). JBOPFO 1083-3668 10.1117/1.JBO.20.6.066005 26065839 PMC4463032

[33] Tseng S. Grant A. Durkin A. J., “In vivo determination of skin-infrared optical properties using diffuse optical spectroscopy,” J Biomed Opt. 13, 1–15 (2008). 10.1117/1.2829772 PMC2626348 18315374

[34] Prahl S., “Everything I think you should know about inverse adding-doubling,” http://omlc.org (2011).

[35] Nomoni M. May J. M. Kyriacou P. A., “Novel polydimethylsiloxane (PDMS) pulsatile vascular tissue phantoms for the in-vitro investigation of light tissue interaction in photoplethysmography,” Sensors 20, 1–13 (2020). SNSRES 0746-9462 10.3390/s20154246 PMC7435705 32751541

[36] Falanga V. Bucalo B., “Use of a durometer to assess skin hardness,” J. Am. Acad. Dermatol. 29, 47–51 (1993). 10.1016/0190-9622(93)70150-R 8315077

[37] May J., Investigation of Fontanelle Photoplethysmographs and Oxygen Saturations in Intensive Care Neonates and Infants Utilising Miniature Photometric Sensors, City University of London (2013).

[38] ams-OSRAM AG, “OSRAM SFH 7016 datasheet,” https://look.ams-osram.com/m/1a73f8d425cfcc40/original/SFH-7016.pdf (2022).

[39] Wang L. Jacques S. L. Zheng L., “MCML–Monte Carlo modeling of light transport in multi-layered tissues,” Comput. Methods Programs Biomed. 47(2), 131–146 (1995). 10.1016/0169-2607(95)01640-f 7587160

[40] Chowdhury M. H. et al., “Estimating blood pressure from the photoplethysmogram signal and demographic features using machine learning techniques,” Sensors 20, 3127 (2020). 10.3390/s20113127 32492902 PMC7309072

[41] Elgendi M. et al., “Recommendations for evaluating photoplethysmography-based algorithms for blood pressure assessment,” Commun. Med. 4, 140 (2024). 10.1038/s43856-024-00555-2 38997447 PMC11245506

[42] Charlton P. H. et al., “Assessing hemodynamics from the photoplethysmogram to gain insights into vascular age: a review from VascAgeNet,” Am. J. Physiol. Heart Circ. Physiol. 322, H493–H522 (2022). 10.1152/ajpheart.00392.2021 34951543 PMC8917928

[r43] Mcveigh G. E. et al., “Age-related Abnormalities in arterial compliance identified by pressure pulse contour analysis aging and arterial compliance,” http://www.hypertensionaha.org (1999). 10.1161/01.hyp.33.6.1392 10373222

[44] Allen J. Murray A., “Age-related changes in the characteristics of the photoplethysmographic pulse shape at various body sites,” Physiol. Meas. 24, 297–307 (2003). PMEAE3 0967-3334 10.1088/0967-3334/24/2/306 12812416

[45] Setchfield K. et al., “Relevance and utility of the in-vivo and ex-vivo optical properties of the skin reported in the literature: a review [Invited],” Biomed. Opt. Express 14, 3555 (2023). BOEICL 2156-7085 10.1364/BOE.493588 37497524 PMC10368038

[46] Kyriacou P. Chatterjee S., “The origin of photoplethysmography,” in Photoplethysmography: Technology, Signal Analysis and Applications, Kyriacou K. A. Allen J., Eds., pp. 17–42, Elsevier (2021).

[47] Al-Halawani R. Chatterjee S. Kyriacou P. A., “Monte Carlo simulation of the effect of human skin melanin in light–tissue interactions,” in Proc. Annu. Int. Conf. IEEE Eng. Med. Biol. Soc., EMBS Vols 2022-July, Institute of Electrical and Electronics Engineers Inc., pp. 1598–1601 (2022). 10.1109/EMBC48229.2022.9871350 36085750

[48] Chan Y. T. N. Rathod M. Franklin D., “Skin tone corrected pulse oximetry models evaluated through reflective pulsatile Monte Carlo simulations,” Biophotonics Disc. 2, 032507 (2025). 10.1117/1.BIOS.2.3.032507 PMC13052488 42028253

[49] Prahl S., “Optical absorption of hemoglobin,” Oregon Medical Laser Center, https://omlc.org/spectra/hemoglobin/ (1999).

[50] Rea M. S. Bierman A., “Light source spectra are the likely cause of systematic bias in pulse oximeter readings for individuals with darker skin pigmentation,” Br. J. Anaesth. 131, e101–e103 (2023). 10.1016/j.bja.2023.04.018 37188559

[51] Vogt W. C. Wear K. A. Pfefer T. J., “Phantoms for evaluating the impact of skin pigmentation on photoacoustic imaging and oximetry performance,” Biomed. Opt. Express 14, 5735 (2023). BOEICL 2156-7085 10.1364/BOE.501950 38021140 PMC10659791

[52] Swamy S. K. N. et al., “Pulse oximeter bench tests under different simulated skin tones,” Med. Biol. Eng. Comput. 63, 1931–1942 (2024). MBECDY 0140-0118 10.1007/s11517-024-03091-2 38653879 PMC12204950

